# Chimeric mis-annotations of genes remain pervasive in eukaryotic non-model organisms

**DOI:** 10.1186/s12864-025-11765-w

**Published:** 2025-07-01

**Authors:** Andreas Bachler, Thomas K. Walsh, Rahul V. Rane, Gunjan Pandey

**Affiliations:** 1 CSIRO, Black Mountain Laboratories, Clunies Ross Street, Canberra, ACT 2601 Australia; 2 CSIRO, 351 Royal Parade, Parkville, VIC 3052 Australia

**Keywords:** Genome annotation, Non-model organisms, Chimeric mis-annotations

## Abstract

**Background:**

Accurate annotation of protein-coding genes is critical for genome analysis in non-model organisms. However, limited RNA-Seq data and incomplete protein resources can lead to errors, including chimeric gene mis-annotations, where two or more adjacent genes are incorrectly fused into a single model. These errors often persist due to annotation inertia, where mistakes are propagated and amplified through data sharing and reanalysis, and leads to cases where the mis-annotated model becomes favoured over the correct model. This complicates almost all downstream genomic analyses such as gene expression studies and comparative genomics.

**Results:**

We investigated chimeric mis-annotations across 30 recently annotated genomes spanning invertebrates, vertebrates, and plants, identifying 605 confirmed cases. The majority of these errors occurred in invertebrates and plants. Using structural prediction and splicing assessment, we demonstrated that utilising machine-learning annotation tools (such as Helixer) provides an approach which can identify mis-annotations.

**Conclusions:**

This study highlights the prevalence of chimeric mis-annotations in genomic datasets and showcases the potential of machine-learning tools such as Helixer to refine gene models for highly variable gene families with mis-annotations present in databases. By addressing these annotation errors, we improve genomic data reliability and facilitate a deeper understanding of non-model organisms.

**Supplementary Information:**

The online version contains supplementary material available at 10.1186/s12864-025-11765-w.

## Background

Annotating protein-coding genes in eukaryotic non-model organisms remains a significant challenge in genomics [1]. Improvements in sequencing technology have dramatically increased the number of high-quality de novo genomes available for many eukaryotic organisms. Yet deriving meaningful insights from these genomes relies heavily on accurate annotation, particularly of protein-coding genes. Producing reliable gene models ideally utilises extensive data, including RNA sequencing and proteomic information from multiple time points, and tissue types. These multiple forms of evidence are then evaluated and result in a summary of the information available for the structure of a gene in the form of coordinates of exons and coding sequences on the genome [2]. For non-model organisms, however, such data are often limited, making it necessary to draw on evidence from closely related organisms. Consequently, genome annotations frequently depend on extrapolated data, which can introduce errors that are challenging to detect and correct-especially when only the final annotations, without supporting evidence, are available.

A possible type of annotation error is a ‘chimeric’ gene mis-annotation, where two (or more) distinct adjacent genes are mistakenly merged as one gene. Chimeric annotations are prevalent in eukaryotic genomes, particularly those with complex splicing patterns, as genome annotation programs, and pipelines face challenges in accurately discerning which genomic regions contribute to a single gene’s coding sequence [3, 4]. These longer, chimeric mis-annotated genes, once established in databases, frequently serve as evidence for annotating newer genomes, and the mis-annotation perpetuates and amplifies. Chimeric genes, due to their larger size, often have higher sequence alignment scores in local alignments like BLAST, leading them to be more likely to be retained than smaller, ‘correct’ alignments. The impact of chimeric gene annotations is broad, from incorrect interpretation of the number of genes in gene families for comparative genomics to incorrect expression profiles for genes in differential gene expression analysis. The inclusion of mis-annotated models in published research poses a particular challenge for scientific reproducibility, as subsequent studies using updated genome versions may reach contradictory conclusions despite improvements in underlying sequence quality. This problem is especially acute in comparative genomics, where conclusions about gene family evolution or function may be fundamentally altered by the presence of chimeric mis-annotations in even a small number of species.

For researchers focused on non-model organisms the availability of reference genome databases is crucial. General genome repositories, like RefSeq [5] or Ensembl [6], provide an invaluable resource for consistent and centralised data to use in comparative analysis or to aid in interpretation of genomic data from their target organism. Reference databases specific to certain organisms or groups, like Phytozome for plants [7], WormBase for nematodes [8] or FlyBase for Drosophila [9], can provide more specialised genome annotation and curation for their target group. Individual organisms can also be annotated by research groups themselves, such as done for *Daucus carota* [10], and can be presented alongside annotations, as is done in these circumstances by RefSeq. These reference databases host and curate genomic datasets to a high standard, and, generally when errors are identified they can be addressed appropriately. As part of annotation workflows of new organisms, it is reasonable to rely on previous annotations to enable linkage and incorporation into existing knowledge. However, this can lead to the propagation of mis-annotations in genomic databases if these are not initially identified, reinforcing mis-annotated gene models. This challenge grows as advancements in long-read sequencing have made it possible to generate many high-quality genomes for non-model organisms, with less time available for manual curation from researchers. With multiple genome versions becoming available for the same species, the need for improved tools to evaluate and correct mis-annotations in genome annotations is important.

Recent advancements in genome annotation have leveraged machine learning to attempt to accurately identify protein-coding genes. Tools like Helixer [11] and Tiberius [12] utilise deep learning models trained on reference databases (in the case of Helixer a selection of RefSeq and Phytozome genomes), to generate models that can be used to annotate the protein-coding genes of genomes without extrinsic evidence. The ability to generate generally correct gene models for the genome of an organism, without any extrinsic information except for the selection of a model, is incredibly useful, particularly for producing candidate annotations. Deciding which annotations are reliable and which are artifacts remains a challenge, although incorporation of extrinsic data (like RNA-Seq) is available for Helixer to improve reliability.

In this study, we aim to highlight and focus on the problem of chimeric mis-annotations for non-model eukaryotic organisms. While tools such as Helixer show promise, their ability to reliably annotate protein-coding genes can be limited when applied to non-model organisms that differ significantly from their training datasets. This study addresses this gap by applying Helixer to a sample of non-model organism genomes and providing a simple validation procedure, that uses a trusted protein dataset to validate the Helixer annotations and to help identify mis-annotations. We demonstrate that many chimeric mis-annotations stem from previously propagated errors from non-curated datasets, highlighting the importance of proactive identification and correction. This will be particularly valuable as high-quality genomes for a broader range of species continue to be generated at an increasing pace, ensuring that errors are identified and addressed before they can be perpetuated through subsequent annotation efforts.

## Results

### Assessment of recent non-model genome annotations to identify chimeric gene models

We developed a systematic validation procedure that leveraged Helixer annotations and a high-quality protein dataset to identify annotations where support for the Helixer gene models was higher than the reference genome models (see Supplementary Fig. 1). For each candidate mis-annotated gene we then performed manual inspection to determine whether each reference gene model should be classified as chimeric or not based on the available evidence from RefSeq’s gene viewer. For each gene model we categorised it as either: (1) “chimeric,” where the data supported that the gene model likely represented multiple distinct genes; (2) “not chimeric,” where evidence supported the RefSeq gene model as a single gene; or (3) “unclear,” where available evidence was insufficient to classify the gene model. This procedure was applied across 30 genomes including invertebrates, vertebrates, and plants (which are the annotation models currently available for Helixer).

**Fig. 1:**
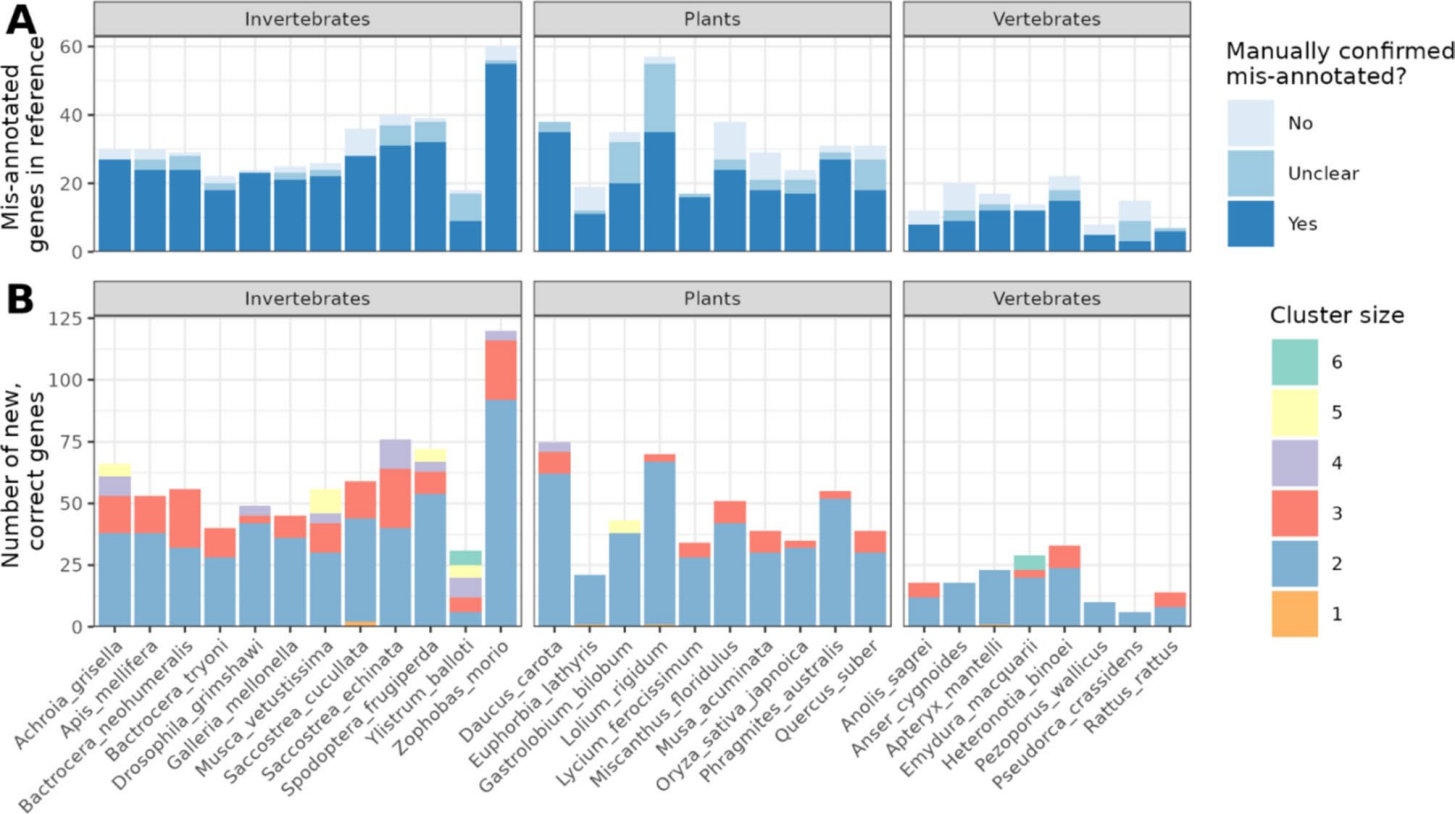
Distribution of mis-annotated chimeric genes in the assessed reference genomes from RefSeq. The candidate chimeric genes were identified from comparison of the reference annotation to that produced by Helixer and assessed for validity to the high-quality SwissProt database. Each potential chimeric candidate gene was then manually assessed for lack of splice support in the RefSeq GeneViewer. **A** The total number of confirmed, unclear or not mis-annotated genes in each reference assessed. **B** The total number of component genes present in the mis-annotated genes identified in each organism. Yes = confirmed mis-annotation; Unclear = evidence not sufficient for designation; No = confirmed correct gene model in reference annotation

From our manual inspection of the candidate chimeric gene models, a total of 605 genes were confirmed as chimeric mis-annotations, while 101 genes were determined not to be chimeric (Fig. 1A). For 107 genes, the available evidence was insufficient to make a definitive classification. The occurrence of chimeric mis-annotations varied across taxonomic groups. The invertebrate group (number of genomes = 12) showed the highest number of mis-annotated genes, with 314 chimeric cases, followed by plants (number of genomes = 10) with 221 cases, and vertebrates with the lowest count at 70 cases (number of genomes = 8). Chimeric mis-annotations were generally cases of two genes being mis-annotated as a single gene (*n* = 499), although there were cases where the number of genes in the chimeric mis-annotation were higher, for example there were 81 genes composed of a chimera of three genes, and 20 genes that were composed of four or more genes (four-mers = 12, five-mers = 6, six-mers = 2) (Fig. 1B). In 5 cases Helixer only produced a single candidate gene model, indicating potentially just a mis-annotated gene. Across the confirmed mis-annotated genes, Helixer produced a total of 1,336 gene models, offering an alternative representation of these mis-annotated regions which more closely aligns with the protein evidence from the SwissProt database.The list of the mis-annotated proteins is available in Supplementary Data File S1.

### Characterisation of mis-annotated chimeric genes

**Fig. 2:**
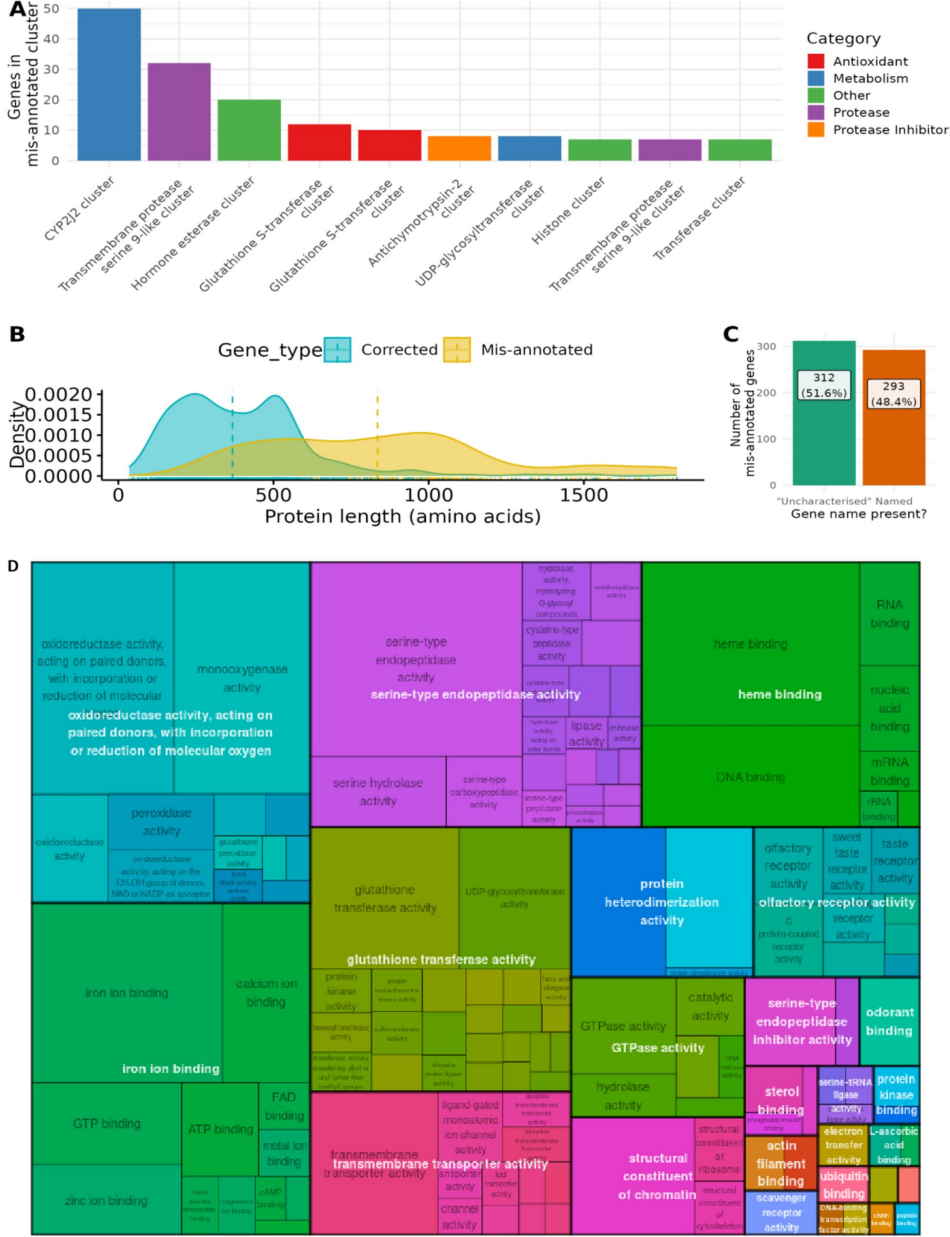
Characterising mis-annotated chimeric genes. **A** Mis-annotated genes were clustered based on sequence similarity using mmseqs easy-cluster and the largest 10 clusters shown. **B** The length of the protein sequences for the longest isoform for each of the confirmed mis-annotated gene models and the Helixer (‘corrected’) gene models is shown **C** The proportions of mis-annotated genes which were labeled as ‘uncharacterized’ **D** TreeMap of summarised Molecular Function GO terms for mis-annotated genes. GO terms were derived for each protein sequence using InterProScan. The treemap was generated using rrvgo

To better understand the source and nature of chimeric mis-annotations, the mis-annotated proteins were clustered based on sequence identity using mmseqs easy-cluster [13]. Clustering allows us to identify patterns in the mis-annotations, such as whether specific gene families or functional categories are more prone to these errors. The largest cluster was composed of 50 mis-annotated chimeras of the cytochrome P450 CYP2J2, followed by 32 genes, clustering in a group of proteases, 20 for hormone esterase, and finally 12 mis-annotated genes in Glutathione S-Transferases (GSTs). The majority of mis-annotated chimeras identified were in clusters with less than 5 members (Fig. 2A).

Comparing the length of the confirmed mis-annotated genes with the gene annotations from the Helixer annotations reveals that the reference genes are generally larger, with a broad distribution from 500 to 1250 amino acids, with a mild peak ~ 1000 amino acids, while Helixer annotations show for the matching regions show a weak bi-modal distribution with two peaks at ~ 250 amino acids and ~ 500 amino acids (Fig. 2B).

The names of chimeric mis-annotations were more likely to be ‘uncharacterised’ than named (Fig. 2C). Addressing the mis-annotation for these would likely lead to clearer naming and improved functional understanding.

Molecular function GO terms from mis-annotated genes were derived using InterProScan and then summarised using rrvgo [14] to 80% similarity and filtered to remove low frequency GO terms (those occurring less than 2 times). GO term analysis of the mis-annotated genes reveal broad functions impacted by mis-annotated chimeric genes, ranging from those involved in metabolism and detoxification (such as cytochrome P450s and glycosyltransferases) to those involved in DNA structure (histone related), as well as olfactory receptors and iron binding related functions (Fig. 2D). These functions are characteristic of multi-copy gene families which display gene duplication and subsequent functional expansion, and so may be more likely to lead to chimeric mis-annotation (see Supplementary Table S1 for evidence linking GO terms to multi-gene family designation).

### Assessment of the three main mis-annotated gene families in insects

#### Cytochrome P450 mis-annotations in Apis species

In our assessment of cytochrome P450 mis-annotations in *Apis* species we identified multiple cases where distinct P450 genes were incorrectly fused into single models. One example was the gene model LOC412936 (GB49894) in *Apis mellifera*, which incorrectly merged three separate P450 genes into a single annotation. This mis-annotation was consistently observed across other *Apis* genomes, including *A. cerana*,* A. dorsata*, and *A. laboriosa*, suggesting that it has been propagated across multiple reference databases, however in these cases sometimes it was only two adjacent P450s that were merged, with the third being present as a correct, distinct gene. Another similar example is the gene model LOC725621 (GB13748), where two single-exon P450 genes were merged, which again, when checked in other *Apis* species, was always present in its mis-annotated state (~ 1000 amino acids, rather than 2x ~ 500 amino acid sequences).

To further check this mis-annotation in another species, we used long-read RNA-Seq data from *A. cerana* and inspected the alignments of long-read RNA-Seq data against the whole genome at these specific locations. The homologous gene in *A. cerana* for the mis-annotated model from *Apis mellifera* (LOC412936 “Uncharacterized protein”) is LOC108003966 (“Uncharacterized protein”). Visual analysis of the long-read RNA-Seq alignments at this region provided strong support for the existence of distinct P450 genes rather than a single chimeric model, reinforcing that these genes should be separately annotated (see Supplementary Figure S2).

When re-analysing the most variable Cytochrome P450 found in [15] (“CYP6 AS1”), we found that one of the Cytochrome P450 sequences included shows signs of mis-annotation (*Bombus terrestris*, LOC100647549;XP_048267503.1). The authors did also identify this mis-annotation and split the gene into the two component Cytochrome P450s, however they designated these as CYP6AS74 and CYP6AS75.

#### Mis-annotation of Glutathione S-Transferases (GSTs) in *Spodoptera frugiperda* corn and rice strains

Comparative analysis of Glutathione S-Transferase (GST) gene models in *Spodoptera frugiperda* revealed multiple discrepancies between the RefSeq, corn, and rice strain annotations, including distinct instances of chimeric mis-annotations. Initially, the RefSeq annotation of *S. frugiperda* was analyzed, identifying four mis-annotated GST genes (LOC118271633, LOC118261724, LOC118266848, and LOC118270149). For *S. frugiperda*, the corn and rice strains have both been annotated separately to RefSeq by the BioInformatics Platform for Agroecosystem Arthropods (BIPAA) and these genomes and annotations are often used when focusing on these specific strains of *S. frugiperda*. We evaluated the presence of these 4 mis-annotated GSTs from the RefSeq reference in the corn and rice strains using reciprocal best hits and individual protein sequences were structurally analyzed using the AlphaFold3 prediction tool. The Predicted vs. Aligned Error (PAE) plots provide a graphical representation of the likely component structure of these candidate mis-annotated GST genes. In brief, these plots provide the per-atom (summarised to per-residue) confidence scores for the relative positioning of amino acid residues within the predicted protein structure [16]. Each position in the heatmap represents the expected distance error (in Ångströms) between pairs of residues when the predicted structure is aligned to the true structure. In the PAE plots described here, darker regions indicate high confidence (low predicted error) in the relative positioning of residues, while lighter regions suggest lower confidence (higher predicted error). Artificial joining of genes results in ‘blocks’ of confidence in the PAE. Inspection of these ‘blocks’ can provide an estimate of the number of component genes in a candidate mis-annotated gene (see Supplementary Table S4 for summary of each gene and Supplementary Figure S4 for the AlphaFold3 predicted-vs-aligned error plots for each gene).

Among the identified mis-annotations, one case (LOC118270149) was correctly annotated in both the rice and corn strains, suggesting that the RefSeq annotation alone contained the error. In contrast, LOC118271633 exhibited the same chimeric mis-annotation across all three annotations. The most problematic cases, however, involved LOC118261724 and LOC118266848, where the mis-annotations differed between the corn and rice strains, leading to conflicting interpretations of GST gene counts.

#### Serine protease mis-annotations in *Drosophila grimshawi and Drosophila ficusphila*

Analysis of serine protease gene models in *Drosophila* revealed a case of mis-annotation of a gene model in two species, *D. grimshawi* and *D. ficusphila*. Protein BLASTp analysis of the well-characterized *D. melanogaster* serine protease gene CG17012 (Q0GSS5_DROME) identified a homologous but mis-annotated gene in *D. ficusphila* (LOC108092397, accession length twice as long as all other hits). The sequence was found to be mis-annotated as a single gene, rather than two distinct genes. A BLASTp of this gene matched a highly mis-annotated gene identified by the current procedure in *D. grimshawi*, LOC6568965, which was found to be mis-annotated with potentially 7 distinct serine proteases merged as a single gene.

### Chimeric mis-annotations are more common in databases with lower levels of curation

**Fig. 3:**
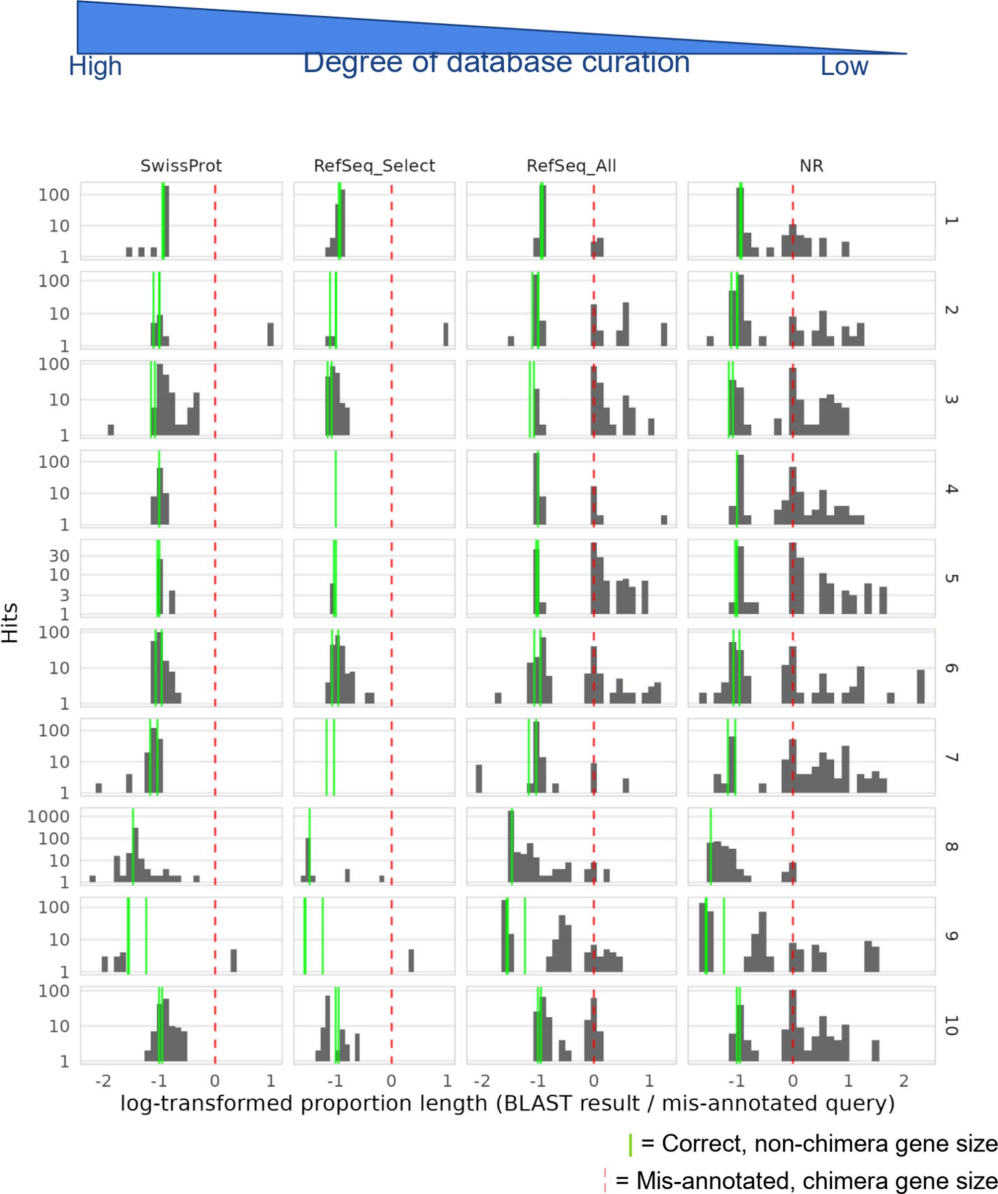
BLAST results of top 10 mis-annotated proteins to four reference databases show more hits in databases with lower curation. Hits filtered for e-values < −30. Green lines indicate Helixer predicted protein size for each mis-annotated chimeric protein. Red line indicates the size of the mis-annotated protein. Log_2_ ratio of length of query to target shown on x-axis (i.e. a hit at −1 indicates that the target hit in the database is half the size of the query). Note y-axis is individually log_10_ scaled for each of the top 10 genes

For the top 10 largest mis-annotated gene clusters, BLASTp analysis against four reference databases reveals a pronounced difference in the amino acid length of the hits between databases with varying curation levels (Fig. 3). Hits were filtered for high-confidence alignments with e-values < 10^–30^. Databases with lower curation levels (such as NR) displaying significantly more hits that match the size of the mis-annotated chimeric proteins, indicating a higher prevalence of these mis-annotations. In contrast, more curated databases like “RefSeq Select” and SwissProt generally align well with Helixer-predicted protein sizes, showing fewer instances of mis-annotated proteins at incorrect lengths.

In comparison to “RefSeq Select”, the “RefSeq All” database exhibits extensive propagation of these chimeric gene mis-annotations, with certain mis-annotated gene clusters, such as gene cluster 3 in Fig. 3, present across hundreds of genomes. These results underscore that chimeric mis-annotations are more frequently observed in less-curated databases, potentially perpetuating these errors across non-model organism annotations.

### Assessment of the most common mis-annotated chimeric gene

**Fig. 4:**
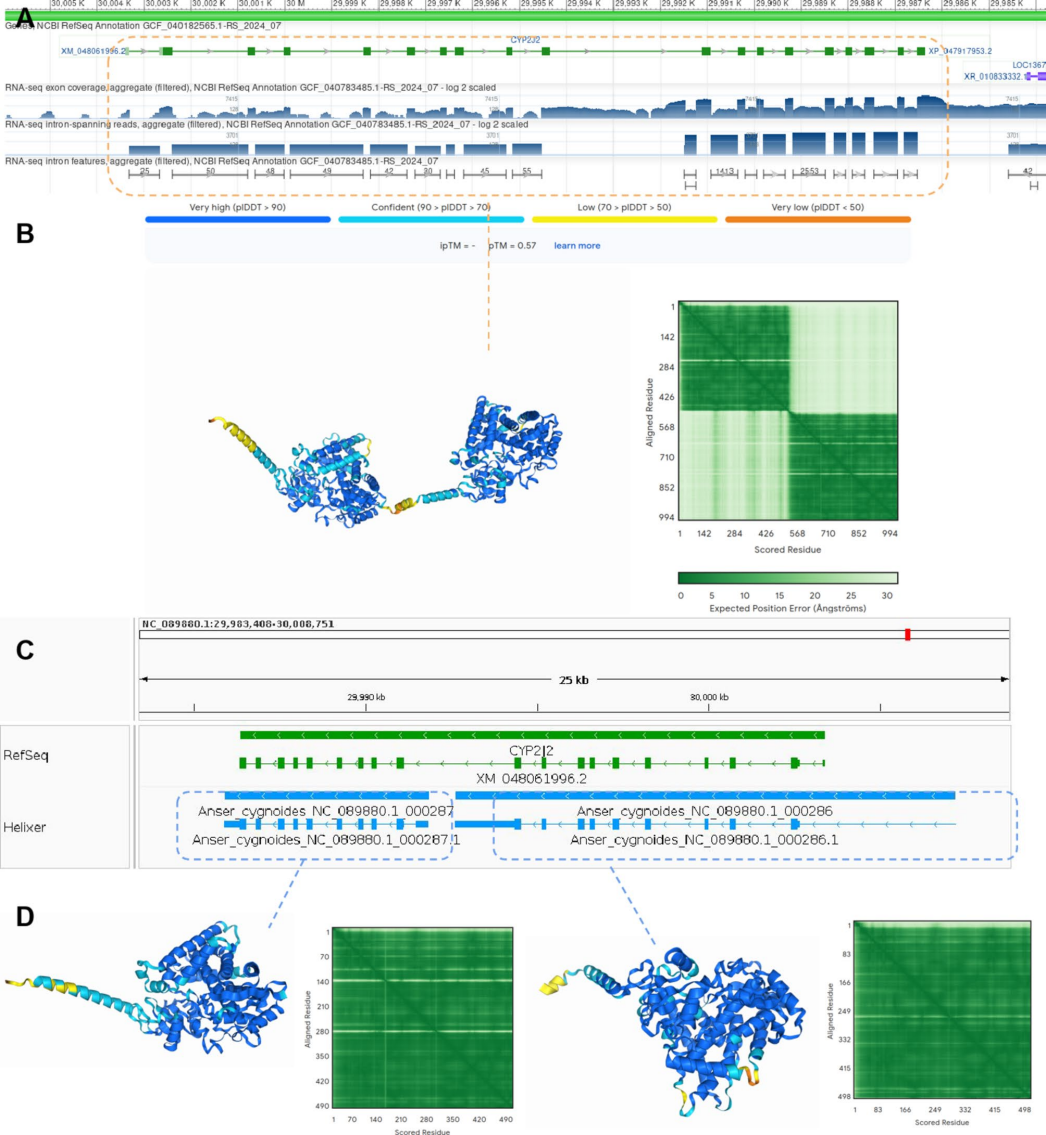
Example of the top mis-annotated chimeric gene in the current study. **A** The gene structure as generated by RefSeq showing RNA-Seq alignment and splice track (CYP2J2 in *Anser cygnoides*; XM_048061996.2/XP_047917953.2) **B** The AlphaFold3 prediction of the protein structure for this sequence using the amino acid sequence of the CYP2J2 gene. **C** Comparison of the RefSeq annotation track and Helixer annotation for this area (note reversed orientation in relation to the structure shown in **A**)) **D** The AlphaFold3 predictions of the protein sequences for the two Helixer annotations

One of the most prominent examples of chimeric mis-annotation identified in the current analysis was the CYP2J2 gene in the latest release of the *Anser cygnoides* (Goose) genome (Gene symbol: CYP2J2, gene ID: 106032922, transcript: XM_048061996.2, protein: XP_047917953.2) (Fig. 4). Analysis of the RNA-Seq coverage at this locus revealed that there are no spliced reads observed between the left hand and right hand regions of this gene, indicating they may represent distinct gene models. The overall transcription profiles also differ, with lower overall coverage for the left side and higher overall coverage of the right side gene structure. Interestingly, the previous annotation for this species (from 2017) at this region had two distinct genes (LOC106032922 and LOC106033102), which appear to have been now merged in this annotation release.

The protein sequence of this gene was used as input for a structural prediction with the AlphaFold3 web-server. The resulting protein structure shows a distinct drop in confidence of the prediction confidence at the point where the two gene models meet. In addition, the predicted-vs-aligned plot supports two distinct gene models for this protein sequence.

In contrast, the annotation produced by Helixer at this region supports two distinct gene models, in agreement with the RNA-seq splicing seen in the GeneViewer. The protein sequences of the gene models from the Helixer predictions were also used as input for AlphaFold3 structures and show high accuracy throughout the overall structure and full coverage of the predicted-vs-aligned plots. From this evidence the Helixer prediction at this locus as having two genes appears to be the most supported.

A representative protein sequence from the top 10 most mis-annotated proteins was also used as input for AlphaFold3 prediction and the predicted-vs-aligned errors showed similar patterns indicative of mis-annotation (see Supplementary Figure S5).

### Some “uncharacterised” genes in the UniRef50 database display characteristics patterns of mis-annotation

**Fig. 5:**
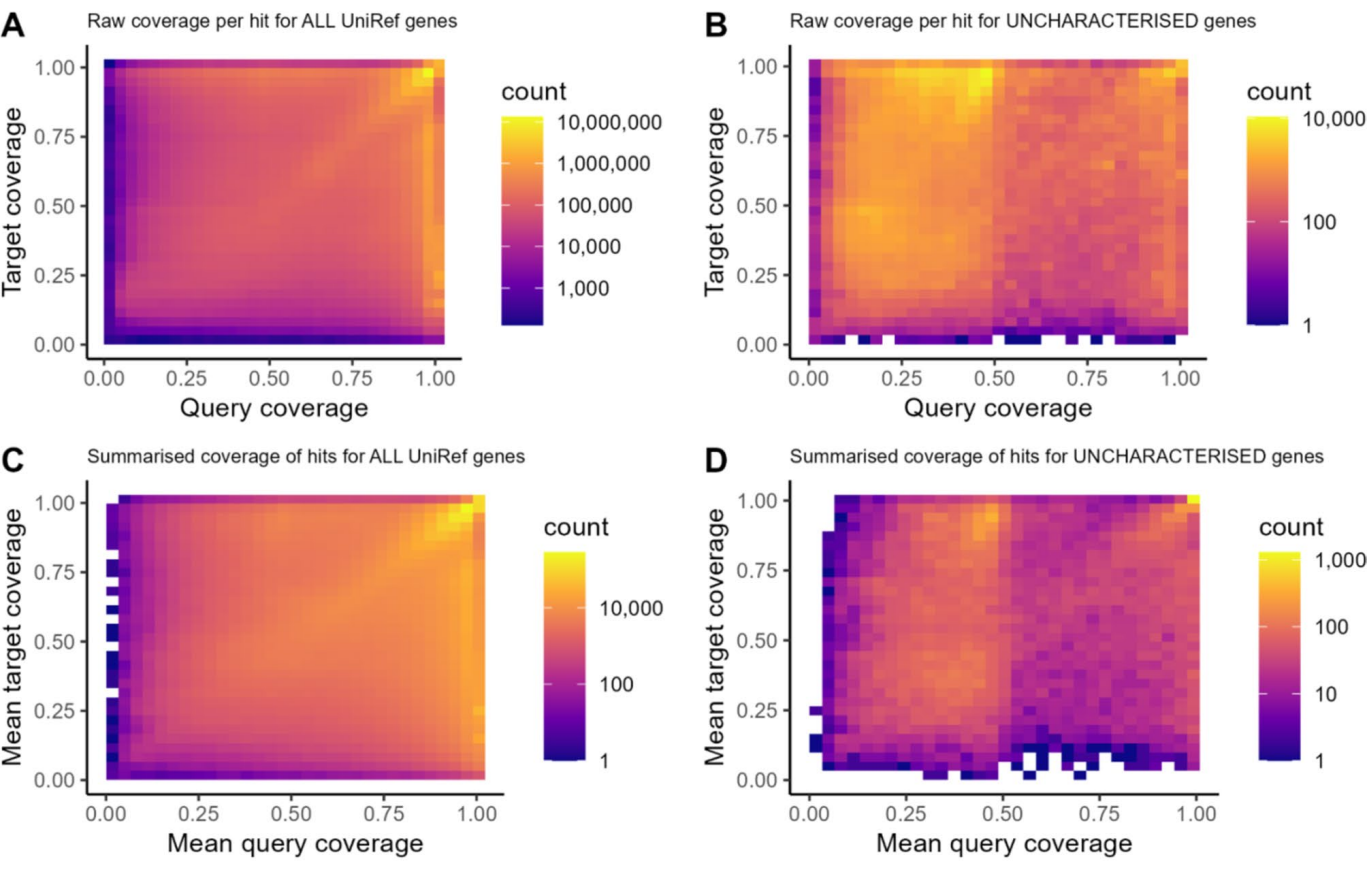
Some ‘uncharacterised’ proteins in the UniRef50 database have characteristic patterns of mis-annotation. The SwissProt database was used as the ‘target’ and the UniRef50 database was used as the ‘query’ for a sequence search using mmseqs easy-search. The UniRef50 database was then filtered to keep only sequences with ‘uncharacterized’ in their name and the same search was conducted with SwissProt as the ‘target’ database. The proportion of query and target coverage for each hit are shown on a 2D binned histogram. **A** and **B** represent the results from all hits above the threshold for the analysed genes. **C** and **D** represent the mean values for the hits when summarised for each gene

Analysis of “uncharacterized” entries within the UniRef databases reveals potential indicators of chimeric mis-annotation. The UniRef databases cluster protein sequences from various sources, including RefSeq and Ensembl, as well as independent submissions, providing only protein sequences without additional annotation details. Mapping high-confidence protein sequences from SwissProt to the UniRef50 dataset and examining the coverage proportions of hits allows for the detection of mis-annotation signals similar to those observed in our filtering procedure. When running with the SwissProt database as the target and the complete UniRef50 database as the query, the hits show a relatively uniform distribution of coverage proportions. However, a distinct pattern emerges when filtering for “uncharacterized” protein sequences, where hits frequently display a difference in coverage between the query and target. In these cases, the SwissProt hits exhibit coverage near 100% coverage, while UniRef sequences display closer to 50%, suggesting potential chimeric mis-annotation (Fig. 5). This discrepancy highlights a potential mis-annotation signal specifically prevalent among “uncharacterized” proteins within the UniRef50 dataset.

### Comparison of the current mis-annotation identification procedure with other datasets

For the RefSeq genome of *Daucus carota* (DH1 v3.0; GCF_001625215.2) an alternate annotation was available on the RefSeq gene viewer, generated by the CarrotOmics/INSDC group as part of the genome project for this organism [10]. The availability of this external annotation provided an opportunity to compare the RefSeq annotations with those produced by an independent research group. Using the Helixer-based validation procedure, we identified 38 candidate chimeric mis-annotations in the RefSeq annotation (Table 1). Manual inspection confirmed 35 of these as genuine mis-annotations. Of these confirmed cases, the CarrotOmics annotation aligned with our findings in 34 instances, correctly separating the chimeric genes into distinct entries. In one case (LOC108212322), our procedure flagged the gene as a chimeric mis-annotation, however both the RefSeq and CarrotOmics annotations treated this as a single gene.

**Table 1:** Comparison of chimeric gene mis-annotations identified by the current Helixer-based procedure and CarrotOmics annotation in *Daucus carota*

Annotation Comparison	Genes	Notes
Candidate mis-annotations identified by current procedure	38	Initially flagged by the Helixer-based procedure.
Confirmed mis-annotations (manual inspection)	35	Verified as chimeric mis-annotations after manual review. Remaining 3 were ‘unclear’ as there was insufficient RNA-Seq coverage to infer splicing.
Agreement of chimeric designation with CarrotOmics annotation	34	CarrotOmics annotation conforms with Helixer-based procedure designation of chimeric gene in 34 out of 35 confirmed cases.
Disagreement case (LOC108212322)	1	Both RefSeq and CarrotOmics annotated this as a single gene, though flagged as chimeric by current procedure.

This collection of annotations for the same genome also provides the opportunity to assess the extent to which Helixer may be prone to generating erroneous fusion mis-annotations (i.e. cases where Helixer predicts a single gene at a locus while the RefSeq annotation has these as separate genes). To evaluate this we found the Reciprocal Best Hits (RBHs) between the RefSeq and Helixer protein sequences, as well as the RefSeq and CarrotOmics protein sequences. Tabulating the cases where the ratios of the best hits aligned to what would be expected for a ‘fusion’ case indicates that Helixer has a generally similar distribution of potential fusions, while the CarrotOmics/INSDC annotation has a much higher ratio of fusions compared to ‘splits’, especially for the simple case of a 1:2 or 2:1 mis-annotation (Supplementary Table S2).

We also attempted to carry out our mis-annotation validation procedure for the genome and annotations analysed previously in which the authors identify and catalogue hundreds of mis-annotated chimeric genes across a collection of vertebrate and invertebrate organisms [3]. The genomes assessed were limited to those for which we could access the same genome assembly and annotation versions used by the researchers, as later annotations had many of the chimeric gene models fixed (assessed genomes described in Supplementary Table S5). Of the 960 total mis-annotated chimeric genes identified by the researchers, the filtering procedure with Helixer identified only 109 cases of mis-annotation (~ 9%), missing 851 mis-annotated genes (Table 2). Please see Supplementary Figure S9 for the distribution of mis-annotations identified between [3] and our method.

**Table 2:** Comparison of mis-annotations identified by [3] with the current validation procedure

	Invertebrates							Vertebrates					Total
	Aea	Bge	Bmo	Eba	Sma	Spu	Tca	Bta	Cmi	Dre	Gga	Xtr	
Total mis-annotations identified by [3]	7	29	56	377	299	9	113	6	14	5	16	29	960
Mis-annotations identified in current procedure	11	0	31	129	91	10	70	6	11	3	7	9	378
Overlap between [3] Mis-annotated gene and the Helixer procedure	0 0%	0 0%	10 17.9%	40 10.6%	33 11%	0 0%	11 9.7%	3 50%	5 35.7%	0 0%	3 18.8%	4 13.8%	109 11.4%
Mis-annotation identified in current procedure but not by [3]	11	0	21	89	58	10	59	3	6	3	4	5	269

Organism codes: *Aedes aegypti* (Aae) - yellow fever mosquito; *Blattella germanica* (Bge) - cockroach; *Bombyx mori* (Bmo) - domestic silk moth; *Episyrphus balteatus* (Eba) - marmalade hoverfly; *Strigamia maritima* (Sma) - centipede; *Strongylocentrotus purpuratus* (Spu) - sea urchin; *Tribolium castaneum* (Tca) - red flour beetle; *Bos taurus* (Bta) - cow, *Callorhinchus milii* (Cmi) - elephant shark; *Danio rerio* (Dre) - zebrafish; *Gallus gallus* (Gga) - chicken; *Xenopus tropicalis* (Xtr) - tropical clawed frog

The Helixer based validation procedure did identify 269 mis-annotated genes which were not identified as chimeric by the authors in [3]. To validate a subset of these novel candidates, we manually examined cases from *Aedes aegypti* for which RNA-Seq alignment data was available for viewing on the online VectorBase portal. We found that some genes, such as AAEL014604 and AAEL014891 (both cytochrome P450s), remain mis-annotated even in the most recent genome versions, although the majority appeared correct. The chimeric cases were further supported by their AlphaFold3 structural predictions (Supplementary Figure S10). To evaluate all of these candidates fully a targeted approach using comparative alignments or RNA-Seq data from each organism might be appropriate, such as we carried out for the case of Cytochrome P450s in *Apis* species detailed previously.

## Discussion

Chimeric gene models remain a significant challenge in eukaryotic genome annotations, primarily due to difficulties in accurately identifying splice junctions and gene boundaries. These errors often result in genes being misclassified as ‘uncharacterized,’ when they are actually cases of incorrect gene fusion. A more systematic approach to identifying chimeric gene annotations could look to identify these errors before they are distributed and lead to difficulties for researchers.

We find that the propagation of chimeric gene models appears to be primarily driven by protein homology evidence in annotation pipelines, rather than specific gene prediction tools or RNA-Seq evidence. Our analysis of the NCBI/RefSeq annotations suggests that when RNA-Seq evidence is available, it often contradicts these chimeric models, indicating distinct genes rather than fused transcripts. However, the weight given to protein evidence in annotation pipelines can lead to the perpetuation of existing mis-annotations from protein databases into new genome annotations. This creates a cycle where incorrect annotations become evidence for future annotations. Another factor contributing to potential mis-annotation is the genome structure and architecture of the target organism, as we find higher incidences in invertebrates compared to vertebrates. Assessing the correlations between various genomic metrics (Supplementary Figure S8) we find that genomes that are smaller, have lower GC% and especially those that have higher gene density (both mean and median intergenic distance, Supplementary Figure S9) are more correlated with mis-annotations in our analysis. This aligns with what has been described in previous approaches for attempting to resolve annotations in complex genomic loci [16]. While it’s challenging to determine the original source of these chimeric models, the persistence of these errors across multiple species and annotations approaches suggests that these factors might be important to consider, for example tuning mapping parameters to be more stringent in organisms or taxa with higher gene density. Continuing to focus on reducing these errors is important, particularly as multiple high-quality genome assemblies become available for diverse eukaryotic organisms [17].

Despite the total number of mis-annotations we identify here per genome being relatively small - dozens or hundreds per organism - the persistent occurrence of such errors is concerning. In our current approach, we utilize Helixer to generate candidate genome annotations for a broad taxonomic range of organisms. Despite being trained on the RefSeq and Phytozome databases (which includes known mis-annotated gene models) in some cases Helixer does not appear to re-generate the mis-annotated chimeric gene models, even when present directly in it’s training data. For example, two of the genomes in the current study happened to be part of the training dataset for Helixer (*Bactrocera tryoni* and *Rattus rattus*), however in both cases there were characteristic chimeric mis-annotations able to be identified by Helixer. This result suggests that Helixer’s model architecture has learned, for some cases, to recognize genuine gene boundaries and splice patterns, even when some of its training examples contain errors, allowing it to generate correct separate gene predictions rather than reproducing chimeric models present in its training data. While this ability to potentially improve upon training data in specific cases of chimeric genes is noteworthy, it’s important to note that not all Helixer annotations are necessarily correct, and it can generate incorrect gene models as well (see Supplementary Table S2). Its outputs need to be carefully validated, such as we demonstrate with the use of a high-quality protein dataset. This represents a promising direction for machine learning approaches in genome annotation, though currently only Helixer has been demonstrated to have this capability across a broad taxonomic range, with other tools like Tiberius being optimized for specific taxonomic groups. While machine learning models so far cannot fully replace genome annotations approaches based on extrinsic evidence, they may serve as a valuable starting point, or like in the current approach for validating genome annotations, akin to the role of Hidden Markov Models (HMMs) used by tools like Augustus [18].

Chimeric gene mis-annotations have wide-ranging impacts across many areas of genomic research. In comparative genomics, such mis-annotations can distort gene family relationships by incorrectly treating chimeric genes as single entities. This misclassification leads to erroneous inferences about gene family evolution. In differential gene expression analyses, chimeric gene models obscure results by averaging the expression signals of their constituent genes, as reads mapped within the mis-annotated boundaries fail to reflect true expression profiles. The common practice of selecting the longest transcript as gene’s representative isoforom exacerbates this issue. An alternative approach, such as the method described by OMark, may help reduce this risk by selecting isoforms with the strongest support relative to known gene families [19]. While this represents an improved approach for selecting representative isoforms, issues with mis-annotations in protein databases, like UniProt and UniRef, persist, as the majority of sequences come from genome annotations. The UniRef cluster process has an explicit step in which larger sequences are preferred during clustering for representatives, which can propagate mis-annotated models into protein structure prediction tools like AlphaFold, ColabFold or Boltz [20–23]. As highlighted in Fig. 5, certain “uncharacterized” large gene models often exhibit patterns indicative of chimeric mis-annotations. Such biases may compromise the accuracy of structural predictions for impacted protein families.

Of particular concern is the impact that these mis-annotations have on the replication and interpretation of previous studies. As demonstrated in the assessment of the most mis-annotated gene families, the merging of distinct genes into larger models results in them being often labelled as ‘uncharacterised’, and leads to inaccurate analysis. In the case of Cytochrome P450s in bees, the mis-annotation of gene LOC412936 occurred during the most recent genome and annotation workflow, and re-conducting the analysis carried out by [15] with the newest genomes and annotations would lead to different, and in the case of inclusion of the mis-annotated model incorrect, conclusions being determined for which Cytochrome P450 families are undergoing expansion or contraction. This is despite the significant improvements that sequencing technologies provide in the contiguity and reliability of genomes. Similarly, for *S. frugiperda*, comparison of GST genes between the corn and rice strains conducted by [23] included differently mis-annotated GST models, which were provided to CAFE for analysis, and gain and loss of these gene families is incorrect based on the mis-annotations identified here. Finally, even in organisms cloesly related to well-studied model organisms such as *Drosophila melanogaster*, there exist mis-annotations which impact understanding, such as variation in presence of serine proteases in more distant species. A lack of focus on mis-annotations, particularly of multi-copy genes, limits the extent to which previous research can be reliably built upon and confounds conclusions about gene family evolution and function across species.

The relatively low numbers of mis-annotations per genome identified by our approach may be because we require Helixer to generate a correct gene model for a target loci, and also on our reliance on SwissProt, a high-quality curated protein datasets, which we use for assessment of gene models from a target organism. This protein dataset cannot necessarily be mapped to all genes in a genome, leading to large numbers of gene models unable to be assessed. This limitation can lead to an underestimation of chimeric mis-annotations for an organism, while more targeted approaches can often lead to higher numbers of mis-annotations identified. In maize, applying multi-genome alignments and RNA-Seq data identified that 3–5% of gene models were chimeric mis-annotations across three closely related organisms [4]. Meanwhile, in a large comparative transcriptomic analysis, it was required to identify and correct hundreds of chimeric and ‘broken’ mis-annotated genes to lead to valid inference of changes in tissue-specific gene expression [3]. When we ran our approach on the same genomes studied by [3] our approach identified only a subset of the mis-annotations found in [3] (see Table 2), however we were able to detect additional cases that were missed in their analysis. These might represent cases where mis-annotations were present across multiple species, where the comparative approach used to identify mis-annotations may be less effective, and highlights the difficulties in identifying chimeric mis-annotations even when extrinsic data such as cross-species alignments are available. An advantage of our approach is that it only requires a genome sequence and annotation for an organism, without reliance on data specific to an organism, which is sometimes not available. Interestingly [3], also found that invertebrate genomes had higher levels of chimeric mis-annotations in comparison to vertebrates, however they do include many model organisms as part of their vertebrate group (i.e. human, mouse, zebrafish), which have had much higher levels of curation than non-model invertebrates. A focused analysis based on comparative genomics will lead to simpler identification of chimeric mis-annotations, since often gene structure is preserved between closely related species, or even within species. With increasing capabilities to generate multiple genomes within species and growing focus on pan-genomic analyses (where gene contraction or expansion is often central) being aware of gene mis-annotations is crucial. The gene models identified here are generally the clearest cases of mis-annotation. With the addition of the manual curation step we have compiled a high-confidence dataset of mis-annotated genes, spanning many organisms and databases.

While our procedure can be used to identify some cases of mis-annotations, a more suitable approach would be to include a more nuanced splicing assessment step within genome annotation workflows. Some bioinformatic tools, such as MIKADO [16], have been developed to identify the ‘best’ transcript from RNA-Seq assemblies, and include explicit processes to identify and flag chimeric mis-annotations. They were able to process transcripts from multiple sources to resolve highly complex loci composed of many multi-copy genes to their ‘true’ component gene structure, Implementation of such a process into genome annotation workflows would likely address many cases of chimeric mis-annotation where there is sufficient evidence from short-read or long-read RNA-Seq sources to reconstruct the target genes. The increasing availability of long-read RNA-Seq data may lend itself to the application of improving splice-junction analysis for genome annotations [24]. While RNA-Seq data is not always available for every gene model, cases like CYP2J2 in Fig. 4, where sufficient RNA-Seq coverage exists and distinct levels of expression are observed between the two gene models, suggest that evaluating the validity of splice sites across all exons could help resolve potential mis-annotations. An additional consideration might be the development of a score or penalty for a lack of support of a splice site when evaluating a whole gene or transcript gene model, as currently this is not clearly defined. A problem for all of these cases is the requirement for expression of these target genes in RNA-Seq data, which is where our current approach provides some benefit. Potential implementation of an approach like MIKADO, or development of metrics to help with ‘splitting’ gene models in genome annotation approaches should help to address these cases, however re-annotation of all currently existing genomes to fix these instances might be computationally prohibitive.

While challenging, addressing chimeric mis-annotations through manual curation is possible. Ideally, these errors should be detected during the genome annotation process. We emphasize that the genomic resources and processes provided by publicly available databases like RefSeq and Ensembl are generally of high quality and are essential for annotating non-model organisms. Overall, these databases provide accurate gene annotations. Our goal, however, is to draw attention specifically to the issue of chimeric mis-annotations, which are often subtle and frequently overlooked, and their impacts on the reproducibility of genomic findings as wetransition away from reference genomes and towards multiple high-quality genomes for individual organisms. Despite their subtlety, these mis-annotations can have substantial consequences for various forms of genomic analysis, and resolving them is critical for improving the accuracy and reliability of genomics in non-model organisms.

## Methods

### Organism and genome selection

The organisms to assess for mis-annotation were initially chosen from those generated by Commonwealth Scientific and Industrial Research Organisation’s (CSIRO) Applied Genomics Initiative (AGI) (BioProject: PRJNA997766) which had been chosen by RefSeq to be the reference annotation for that organism. This was primarily invertebrates (*n* = 10), followed by plants (*n* = 3) and vertebrates (*n* = 3). The *Apis melifera* genome (GCF_003254395.2) and *Drosophila grimshawii* (GCF_018153295.1) genomes were included to prompt investigation of the genome annotations due to their importance in agriculture [25] and previous focus of improvement on annotations [26], respectively. Due to the low number of vertebrate and plant genomes present, additional species were chosen from the latest released RefSeq reference genomes to make up to a minimum of 8 representatives across all groups (RefSeq releases accessed on 23 July 2024).

### Helixer genome annotation

All assessed genomes were re-annotated using Helixer [11]. This tool leverages deep neural networks for accurate ab initio gene structure prediction of provided genome assemblies, requiring only specification of a model from one of ‘vertebrate’, ‘invertebrate’ or ‘land_plant’. The Helixer tool was trained on RefSeq and Phytozome (Version 13) annotated genomes. Two of the genomes produced by AGI (*Bactrocera tryoni* and *Rattus rattus*) were used in the training and validation of Helixer, but not for testing of their model. For each genome the specified model parameters for the ‘vertebrate’ (model: “v0.3_m_0080”), ‘invertebrate’ (model: “v0.3_m_0100”) or ‘land_plant’ (model: “v0.3_a_0080”) was used. The Helixer version used in this manuscript is version 0.3.2. The intermediate to latest releases of Helixer (currently 0.3.5) have not fundamentally changed the model architecture or results but have focused on improving performance and compatibility. Future releases which fundamentally change the model or training data might change the performance of annotations.

### Curated protein database generation

To generate relevant high quality protein databases for these groups the SwissProt protein database was filtered according to the closest taxonomic level to the lineages used by Helixer for annotation (see Table 3). MMseqs2 (version 15-6f452) [13] was used to download the SwissProt database and then subsequently filtered based on taxonomic level using the filtertaxseqdb command.

**Table 3:** SwissProt database sizes following filtering for relevant Helixer lineage

SwissProt filtered database	Taxonomic level	Helixer matching taxonomic model	Number of proteins in database
Protostomia	33,317	Invertebrates	21,712
Deuterostomia	33,511	Vertebrates	41,744
Plants	33,090	Plants	87,425

### Procedure for identification of potential mis-annotated gene models 

The validation procedure was designed to compare reference gene annotations with Helixer-predicted annotations and use a high-quality protein database to try and identify potential chimeric proteins. The script takes a reference GFF file, an ‘alternate’ GFF file (in our case from Helixer), and a reference FASTA file as inputs. It also requires a trusted protein sequence file for the organism (in the current process this is the relevant SwissProt database, filtered according to the taxonomy used by Helixer for predictions) (see Supplementary Figure S1 for workflow). While specifically focused on Helixer during development of the workflow any valid annotation (including from other machine learning genome annotation programs such as Tiberius) can be provided as the alternate annotation for this workflow, provided that only a single isoform is present for each gene, as this is the default output produced by Helixer.

First, for the reference genome annotation the longest isoform per gene was retained. This was not necessary for Helixer as it only produces the longest isoform for each gene model. The reference and Helixer annotations for a genome are compared using gffcompare (0.12.6) [27] to provide links between similar reference and Helixer annotations. Next, protein sequences are extracted for each gene model and searched against the relevant SwissProt database (version 24-Jul-2024) using MMseqs2 (version 15-6f452) (with default parameters except for ‘-s 7.5’). The results were then processed using a series of filters to try and identify signatures of mis-annotation.

1. Median query coverage of hits for the gene in the reference annotation is < 60% and median of query coverage of hits for the gene/s in the matching Helixer annotation/s is > 70%. These thresholds were chosen based on the expected pattern of chimeric genes: when a chimeric gene composed of two similar-sized genes is aligned to its component parts, it should show approximately 50% coverage, with an additional 10% buffer allowed for size variations. The higher threshold for Helixer annotations ensures we capture cases where the individual gene predictions show strong matches to known proteins.
2. Keep only genes where the Helixer hits are a subset of the reference annotation hits based on the database target hit. This filter helps confirm that the differences between annotations represent genuine cases of gene splitting rather than alignment artifacts or alternative gene predictions.
3. The final filter examines the spatial distribution of hits along the reference gene. Hit coordinates are first shrunk by 10% to reduce noise from overlapping alignments, then clustered using pyranges (0.1.2) [28] to identify distinct regions along the reference gene. Genes are retained if they have at least 2 distinct clusters (indicating distinct regions) and the total length covered of the query gene is > 50%, consistent with the expected pattern of chimeric genes composed of multiple distinct domains or gene regions.

The gene models identified from this simple filtering strategy were then manually inspected on GenBank using the GeneViewer. Identification of a lack of supporting RNA-sequence reads over splice junctions was used to confirm whether a gene was likely to be mis-annotated (‘yes’, see example in Fig. 1). If clear RNA-sequence reads connecting the distinct regions of the gene model were present then the gene model was confirmed as correct and not chimeric (‘no’). If there was no RNA-sequence data available or if there was no clear decision able to be made the gene model was assigned ‘unclear’. The validation procedure and SwissProt databases are available at https://github.com/Andy-B-123/AnnotationSplitter and 10.6084/m9.figshare.28236284.v1.

### Evaluation of potential mis-annotated gene models

The protein sequences from all confirmed mis-annotated gene models from the initial screen above were clustered using mmseqs easy-cluster with default parameters across all assessed genomes. The names of the top 10 largest clusters were assessed for function from results from the online BLASTp portal [29] against the SwissProt database (accessed 1/11/24).

The matching Helixer annotations for all the confirmed mis-annotated gene models were extracted and their sequence lengths compared with their parent mis-annotated gene using seqkit stats (version 2.7.0) [30].

### Functional annotation

For each gene, the protein sequence the functional annotation was assessed using InterProScan (version 5.65) [31] to provide GO terms. Unique GO terms for each protein were retained and summarised using rrvgo (version 1.16) [14] to generate a treemap summarising the molecular function GO terms the mis-annotated genes.

### Assessment of top 10 most mis-annotated gene models in current sequence databases

The top 10 most mis-annotated gene models were searched against 4 databases using the online portal for BLASTp [29]. The databases were ‘Swiss-Prot (swissprot)’, ‘RefSeq Select (refseq_select)’, ‘RefSeq (refseq_proteins)’ and ‘NR (nr)’, in order of highest level of curation to lowest level of curation (all accessed on 1/11/24). The resulting hits for each gene were plotted based on the log transformed ratio of the query length (i.e. the mis-annotated gene) vs. the hit length. The matching length of the Helixer annotation for each mis-annotated gene is indicated in green for each of the top 10 most mis-annotated genes.

### Analysis of the three most mis-annotated gene families in specific insects

#### Cytochrome P450s in Apis species

The *Apis mellifera* genome and annotation (GCF_003254395.2) were evaluated for mis-annotations as described above. The mis-annotated gene LOC412936 was identified as three tandem cytochrome P450 genes which had been merged together. The BEEBASE annotation for *Apis mellifera* (amel_OGSv3.2) has two P450s identified in this region (GB49894 and GB49892), missing the third P450 present in the mis-annotated model. A BLASTp search of the mis-annotated gene (LOC412936) against NR revealed hits for the full length mis-annotated protein (~ 1500 amino acids (a.a)) or the double mis-annotated protein (~ 1000 amino acids) in *Apis cerana* (uncharacterized protein LOC108003966, length = 1043 a.a.), *Apis dorsata* (uncharacterized protein LOC102672612, length = 1013 a.a.) and *Apis laboriosa* (uncharacterized protein LOC122712120, length = 1506 a.a.).

For validation, long-read nanopore RNA-Seq data was downloaded for *Apis cerana* from a previous study [32] (PRJNA1116335) and aligned to the genome using minimap with splicing (‘-x splice’). The long-read data came from extractions of Worker, Drone and Queen bees and had a total size of > 100 million reads with mean size ~ 1kbp. Assessing the gene visually in IGV shows no reads joining the distinct genes in the mis-annotated model (Supplementary Figure S2).

This gene is of interest due to its previous assessment for gain and loss across bee species [15]. In the gene annotation used in that study the gene was correctly assessed as a single cytochrome P450 (GB49894), designated as CYP6 AS7. However, in the most recent *Apis mellifera* genome, the sequence annotated as GB49894 is the mis-annotated protein LOC412936 with three component P450s, which in other Apis species sometimes has two or sometimes has three component P450s.

Specifically assessing the most variable Cytochrome P450 found in [15] (“CYP6 AS1”), the protein sequence for *Apis mellifera* (GB40288; UniParc record UPI00003 C0D3B; 100% amino acid sequence match with current record LOC112939925;NM_001365200.1;NP_001352129.1) was used as in a BLASTp search of all NR. Most of the top results for bees were of the correct length (~ 500 amino acids), however for *Bombus terrestris*, which was assessed in the study, the accession was ~ twice as large as the other accessions (LOC100647549; XM_048411546.1; XP_048267503.1), in both the current (GCF_910591885.1) and previous (GCF_000214255.1; LOC100647670; XM_003400081.3; XP_003400129.2) genome annotations. The Ensembl generated annotations for both genomes has these described as separate Cytochrome P450s (ENSBTSG00005026627 and ENSBTSG00005036496). The authors do split this gene to the two component cytochrome P450s in their analysis (XM_003400081.3 A and XM_003400081.3B) and designate these as CYP6AS74 and CYP6AS75. However, the two Ensembl annotations (protein sequences ENSBTST00005053334.1 and ENSBTST00005051253.1) have the best blast hit as the *A. mellifera* CYP6 AS1 match (NP_001352129.1) and a different Cytochrome P450 (XP_026300534.1), respectively, indicating potentially that the designation from the authors may have been based on incorrect splitting of the sequence.

While not used in the study, the matching Cytochrome P450 in *Apis cerana* was also found to be mis-annotated (LOC108003965; XP_028525629.2), as supported by the Helixer-based procedure, manual assessment of the gene model on RefSeq and lack of long-read splicing between the two distinct gene models (Supplementary Figure S3).

#### Mis-annotation of glutathione S-Transferases (GSTs) in Spodoptera frugiperda corn and rice strains

The *S. frugiperda* genome and annotation (GCF_023101765.2) were evaluated for mis-annotations as described above. Mis-annotations for Glutathione S-Transferases (GTSs) were identified based on SUPERFAMILY analysis using InterProScan (SUPERFAMILY: SSF47616; InterPro domain: IPR036282). The protein sequence from the 4 mis-annotated GST proteins was extracted and uploaded to the AlphaFold3 web-server, with the clustering of the predicted-vs-aligned residues used as an estimate of the number of component mis-annotated genes. Protein sequences from the *S. frugiperda* rice (v3.0) and *S. frugiperda* corn (v7.0) strains were retrieved from the BIPAA database (http://bipaa.genouest.org/is/lepidodb/spodoptera_frugiperda/). The protein sequences of the 4 mis-annotated GST proteins were used as a query against the proteomes of both the rice and corn strains of *S. frugiperda* using mmseqs easy-rbh with default parameters.

#### Serine protease mis-annotations in Drosophila grimshawi and Drosophila ficusphila

The *D. grimshawi* genome and annotation (GCF_018153295.1) were evaluated for mis-annotations as described above. A mis-annotated serine protease gene (LOC6568965) was identified based on SUPERFAMILY analysis using InterProScan (SUPERFAMILY: SSF50494; InterPro domain: IPR009003). The protein sequence of this gene was used for an online BLASTp against the NR database limited to Drosophilidae (taxid:7214) and revealed multiple component trypsin/serine protease domains. Matching this was a hit from *Drosophila ficusphila*, another mis-annotated gene (LOC108092397). A BLASTp analysis of this gene against the NR database limited to Drosophilidae (taxid:7214) provided a match against the *Drosophila melanogaster* gene CG17012.

### Investigation of the Helixer annotation for the most mis-annotated gene

The most mis-annotated gene cluster contained 50 genes of a chimeric cytochrome P450 gene. The gene model (CYP2J2; XM_048061996.2; XP_047917953.2) from the Swan goose (*Anser cygnoides*) was chosen from the cluster for further assessment. The protein sequence was extracted and uploaded to the AlphaFold3 web-server [33] and a structure model generated. The protein sequence of the two Helixer annotations from the Swan genome were also extracted and uploaded to the AlphaFold3 web-server [33] and a structure generated. The protein structures and predicted vs. aligned plots are provided in Fig. 4.

### Assessment of the top 10 most mis-annotated genes using AlphaFold3 protein structure predictions

The top 10 most mis-annotated protein clusters were identified and the representative from the mmseqs clustering step was identified. A representative protein sequence from each cluster was uploaded to the AlphaFold3 web-server [33] and the protein structure generated (accessed 14/11/2024). The predicted vs. aligned error data was downloaded in json format and visualised with a heatmap and provided in Supplementary Figure S4.

### Comparison of gene models between reference annotations and Helixer predictions

To compare gene models between different annotation sources, we first standardized the datasets by selecting only the longest isoform per gene from both RefSeq and CarrotOmics/INSDC annotations, matching the output format of Helixer. Protein sequences were extracted from each annotation set. Reciprocal best hits (RBHs) were identified using MMseqs2 [13] with default parameters, comparing RefSeq proteins against both CarrotOmics and Helixer predictions.

For each RBH pair, we calculated the length ratio between the RefSeq protein and its corresponding hit (CarrotOmics or Helixer), applying a log2 transformation to normalize the distribution. To categorize potential gene model differences, we established the following classification criteria based on log2 ratio ranges:

- “2:1 split”: 0.8 ≤ log2 ratio ≤ 1.2
- “3:1 split”: 1.4 ≤ log2 ratio ≤ 1.7
- “4:1 split”: 1.9 ≤ log2 ratio ≤ 2.1
- “1:2 fusion”: −1.2 ≤ log2 ratio ≤ −0.8
- “1:3 fusion”: −1.7 ≤ log2 ratio ≤ −1.4
- “1:4 fusion”: −2.1 ≤ log2 ratio ≤ −1.9
- “1:1 match”: −0.3 ≤ log2 ratio ≤ 0.3

Cases falling outside these ranges were classified as “other”. These thresholds were selected to allow for minor variations in protein length while maintaining specificity in identifying potential gene splits or fusions. The frequency of each category was then tabulated to assess the distribution of gene model differences between annotation sources.

### Analysis of genome characteristics associated with mis-annotations

To investigate potential genomic features associated with chimeric mis-annotations, we analyzed various genome metrics for each species. Basic genome statistics, including N50, total length, and sequence length distributions, were calculated using seqkit stats with the all-statistics flag (-a). Mean GC content was determined using seqkit fx2 tab.

To assess gene spacing characteristics, we developed a custom Python script to calculate median and mean intergenic distances. For simplification, genes overlapping with other genes were excluded from this analysis, which affected less than 2% of genes across all genomes.

Statistical relationships between genome characteristics and the frequency of chimeric mis-annotations were evaluated using Spearman’s rank correlation coefficient, chosen for its robustness to non-linear relationships and outliers. The correlation analysis included genome assembly metrics (number of sequences, total length, minimum length, average length, maximum length, quartile distributions, and N50), sequence composition (GC content), and gene organization features (mean and median intergenic distances, proportion of overlapping genes). Correlation coefficients were visualized using a color-coded correlation matrix, with significance levels indicated by coefficient values. All statistical analyses were performed in RStudio (R version 4.5.0, RStudio 2024.12.2).

### Assessment of larger protein databases for signature of chimeric mis-annotation

The UniRef50 database (accessed August 2024) was downloaded from UniProt [34]. The UniRef50 database was used as a query and the SwissProt database (version 24-Jul-2024) as the target for a high-sensitivity search (mmseqs easy-search with parameters ‘-e 1.000E-010 --min-aln-len 100 -s 1’). Hits were then filtered to keep only those with more than 200 bit score, and for each hit the coverage of the query and target were visualised using a 2D binned histogram with a log-scale for the number of hits. This was then sub-selected to keep only proteins with the string ‘uncharacterized protein’ in the header (case-insensitive).

## Supplementary Information

Supplementary Material 1.

## Data Availability

Data is provided within the manuscript and supplementary information files. The software developed is available at https://github.com/Andy-B-123/AnnotationSplitter. The databases developed for the software are available at https://doi.org/10.6084/m9.figshare.28236284.v1.
