# Supplementary material for: Chimeric mis-annotations of genes remain pervasive in eukaryotic non-model organisms
Source: BMC Genomics. 2025 Jul 1;26:630. doi: 10.1186/s12864-025-11765-w (PMC12220653; doi:10.1186/s12864-025-11765-w)
Supplement: Supplementary file 1 — Supplementary Material 1. [file 12864_2025_11765_MOESM1_ESM.docx]

# Supplementary Material and Methods


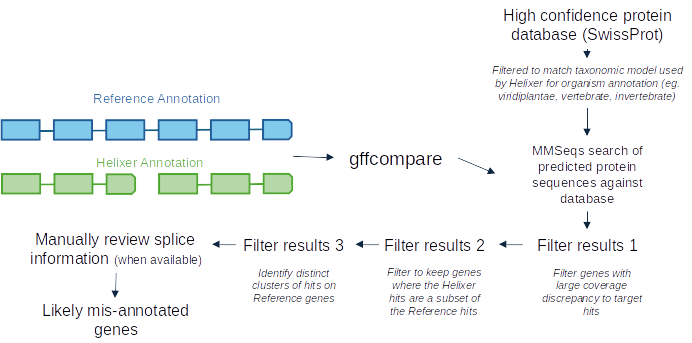


**Supplementary Figure S1. Diagram of the validation procedure for analysing a target genome and annotation for chimeric mis-annotations using Helixer and a trusted protein dataset.**


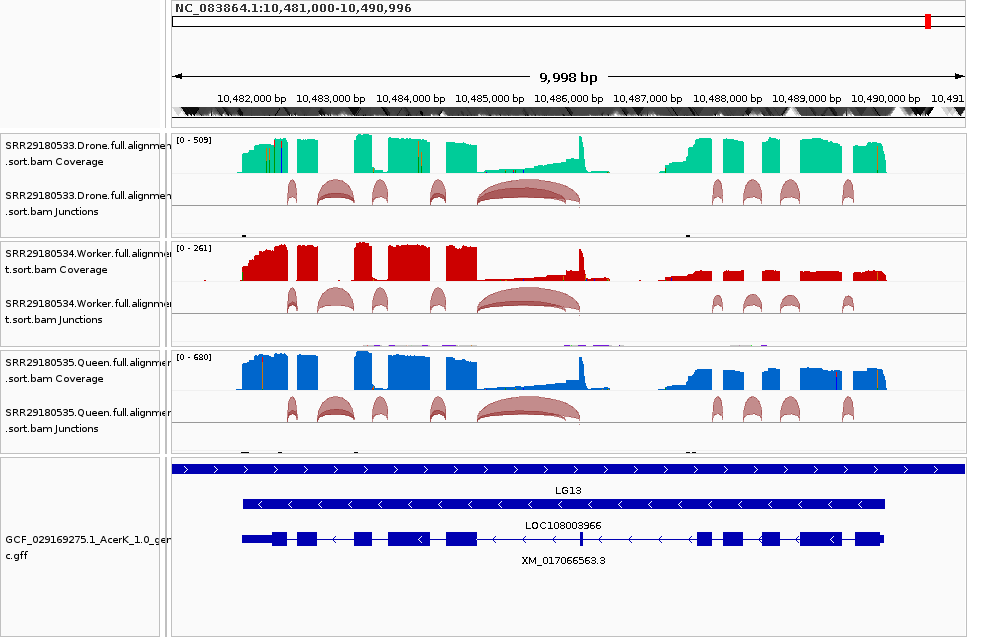


**Supplementary Figure S2. Long-read RNA-Seq alignments for *Apis cerana* show distinct clustering in the mis-annotated Cytochrome P450 gene LOC108003966.** Long-read RNA-Seq data was acquired from PRJNA1116335 and consists of three datasets from a Drone, Worker and Queen bee (SRR29180533, SRR29180534 and SRR29180535, respectively) and aligned to the *A. cerana* reference genome using minimap with the splice option (-x ‘splice’). Alignments were viewed using IGV. Minimum threshold for splice junctions was set to 10 to allow for visualisation, no splice junctions were present across the two distinct genes below this threshold.


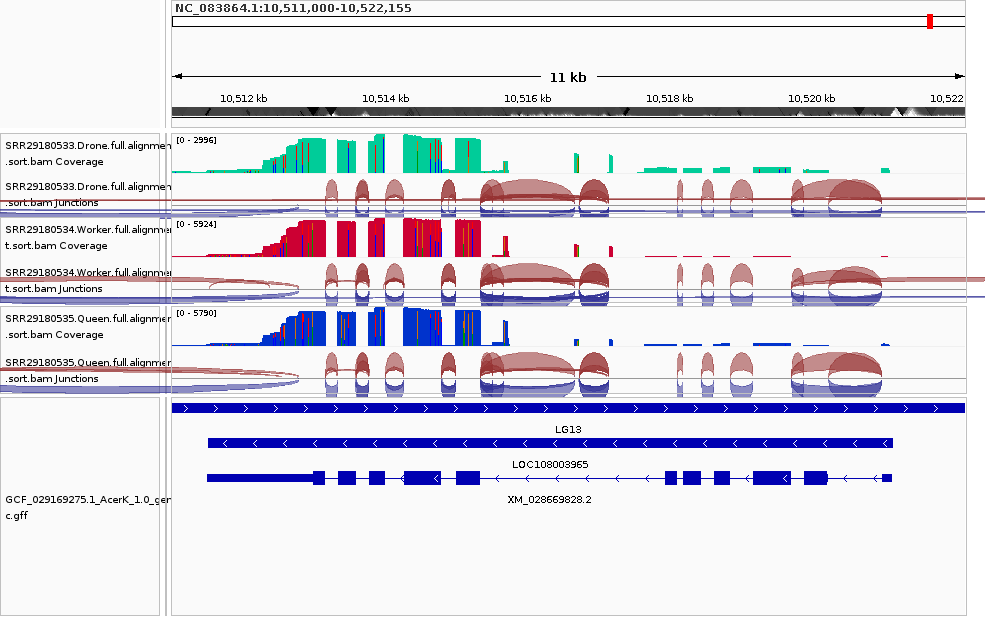


**Supplementary Figure S3. Long-read RNA-Seq alignments for *Apis cerana* show distinct clustering in the mis-annotated Cytochrome P450 gene LOC108003965.** Long-read RNA-Seq data was acquired from PRJNA1116335 and consists of three datasets from a Drone, Worker and Queen bee (SRR29180533, SRR29180534 and SRR29180535, respectively) and aligned to the *A. cerana* reference genome using minimap with the splice option (-x ‘splice’). Alignments were viewed using IGV. Minimum threshold for splice junctions was set to 10 to allow for visualisation, no splice junctions were present across the two distinct genes below this threshold.

**
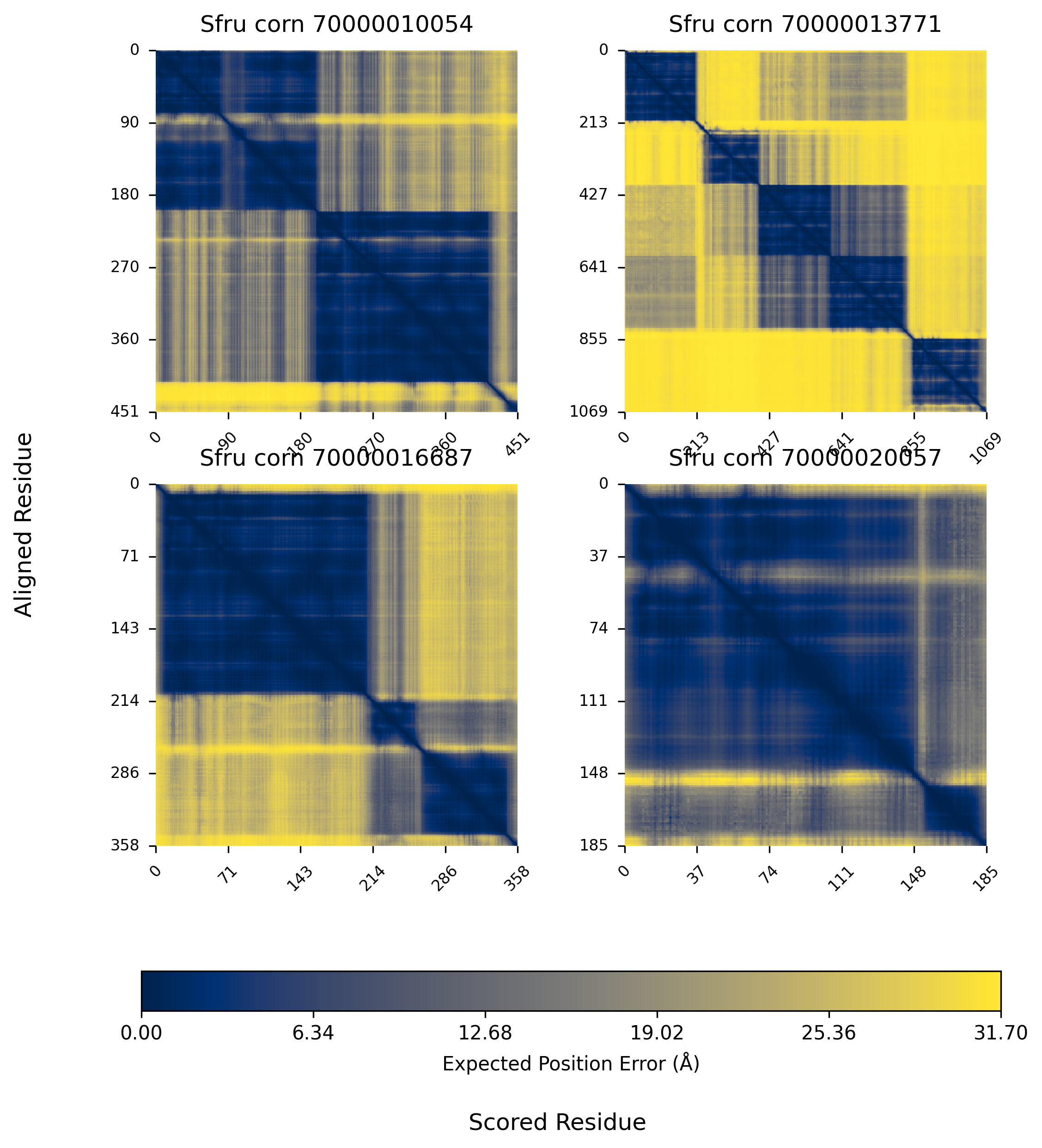

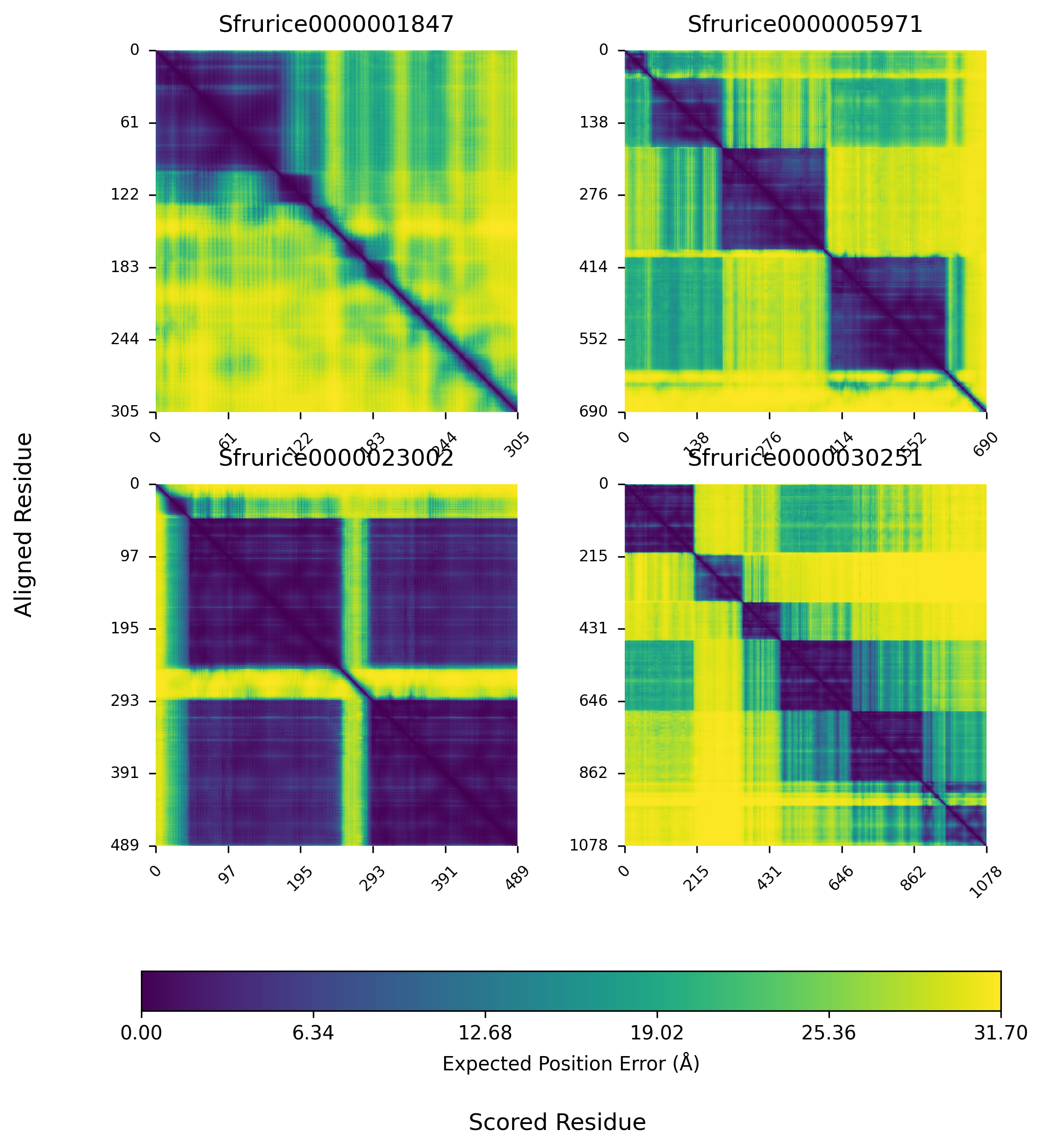
**

**Supplementary Figure S4. AlphaFold3 Predicted vs Aligned Error (PAE) plots for the mis-annotated *Spodoptera frugiperda* Glutathione S-Transferase (GST) genes from the corn and rice strains.**

**
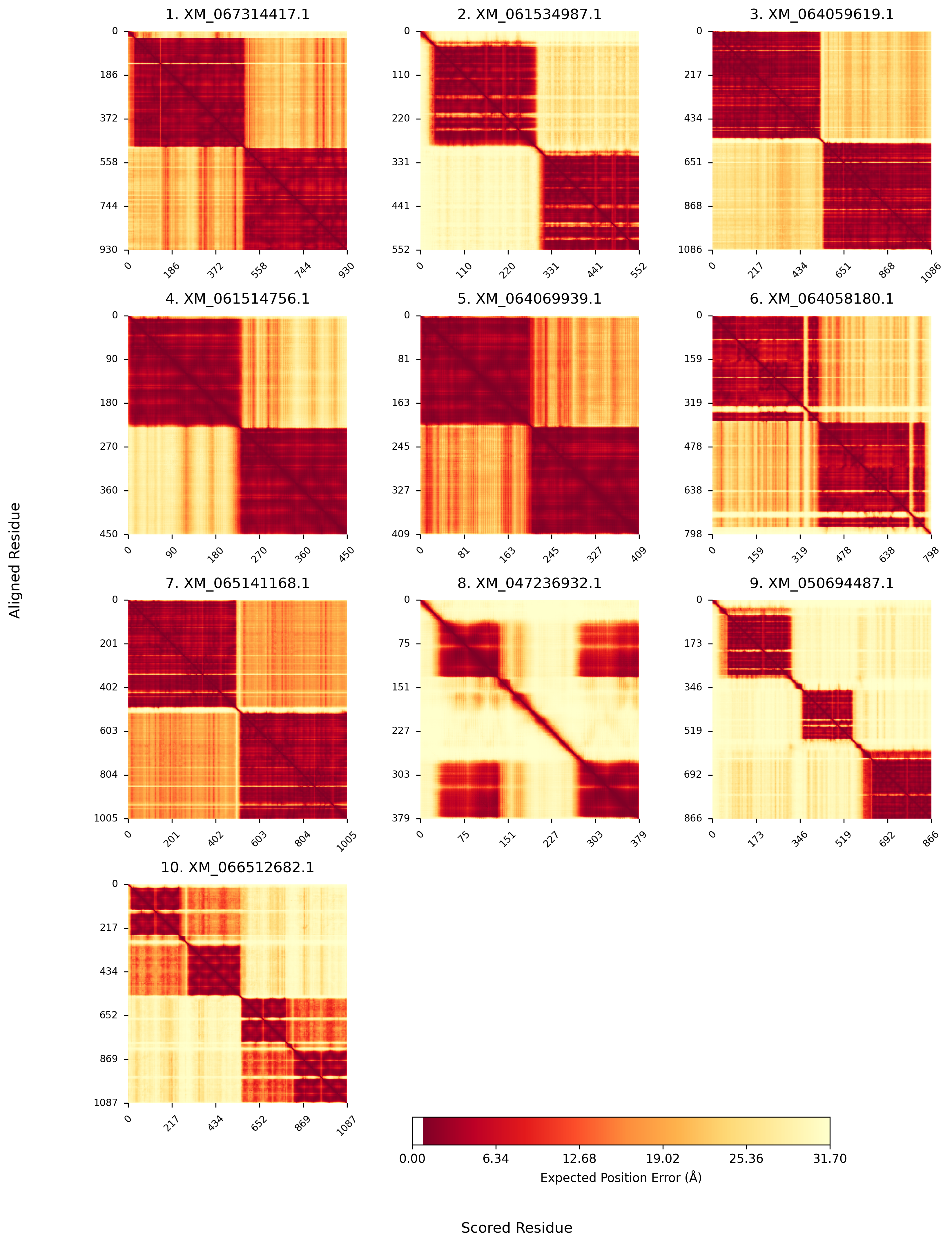
**

**Supplementary Figure S5. AlphaFold3 Predicted vs Aligned errors for the top 10 most mis-annotated genes identified in the current analysis.**

**A)**

**
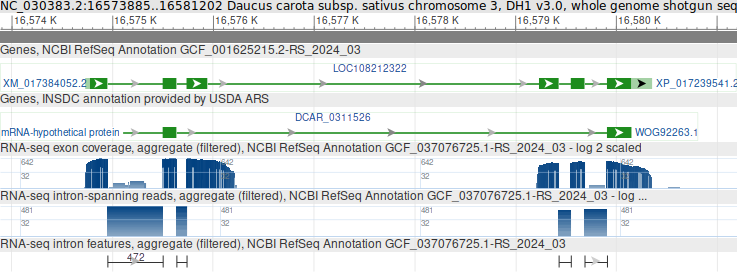
**

**B)**

**
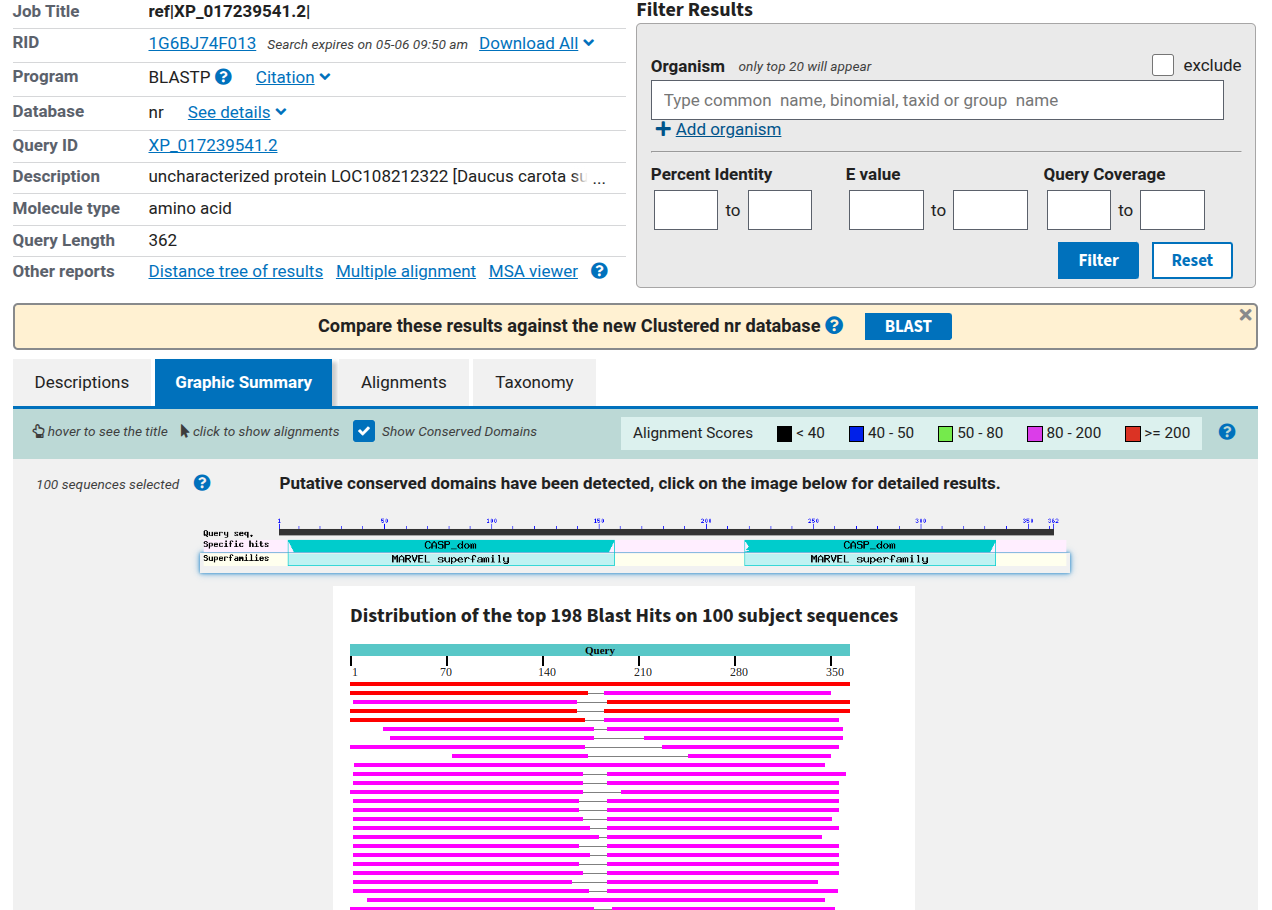
**

**Supplementary Figure S6. Annotation comparison between RefSeq and INSDC/CarrotOmics for LOC108212322 from *Daucus carota.*** A) The two annotation tracks (RefSeq, top; INSDC, bottom) have differences in the exon structure but both annotate this as a single protein. The RNA alignments indicate distinct gene structures, with the exon structure aligning more with the RefSeq annotation. B) This gene was identified as two distinct genes by Helixer, supported by the hits and domains identified using the online BLAST portal for the protein sequence of this gene (XP_017239541.2).


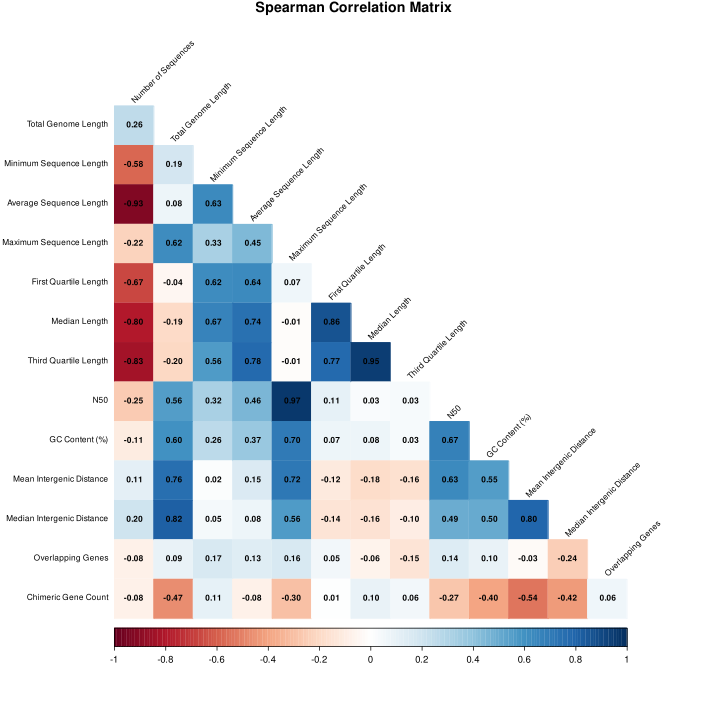


**Supplementary Figure S7. Correlation of various genome metrics with the number of confirmed chimeric genes from each genome.** Highlighted is the row containing the correlations with the number of confirmed chimeric mis-annotations identified in Figure 1 of the main manuscript.


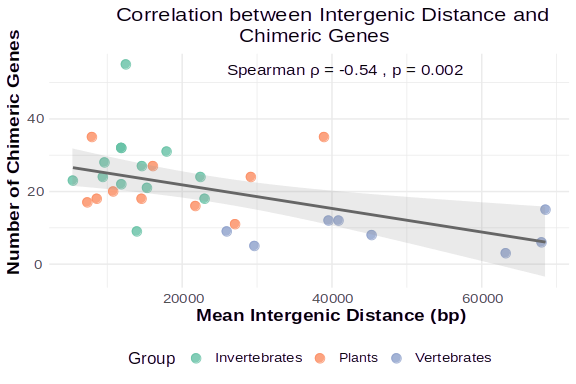


**Supplementary Figure S8. Correlation between mean inter-genic distance and confirmed mis-annotated chimeric genes across the organisms assessed in Figure 1 of the main manuscript.**


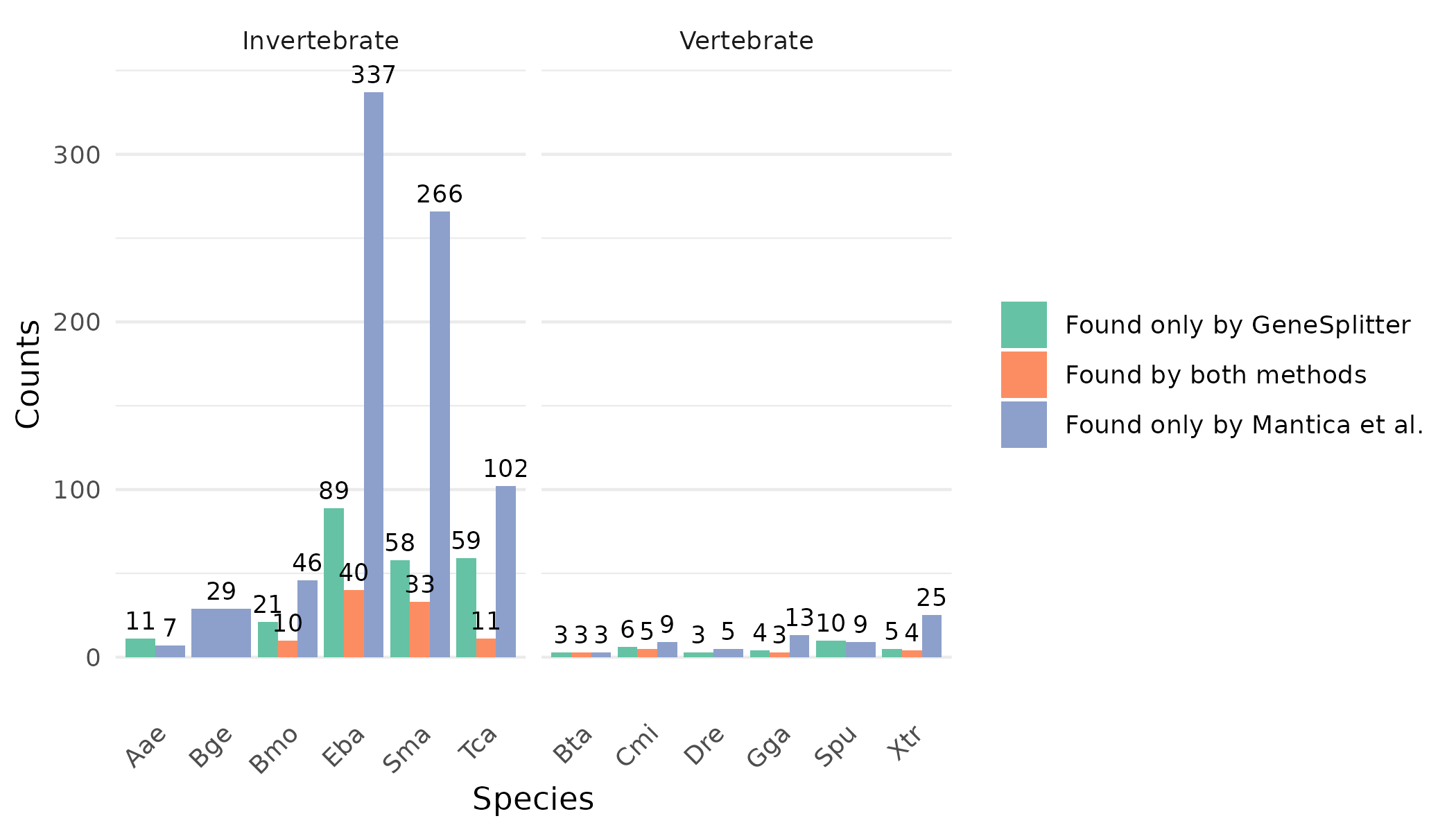


**Supplementary Figure S9. Summary of chimeric cases from Mantica et al [1] and the current process for vertebrate and invertebrate genomes.**


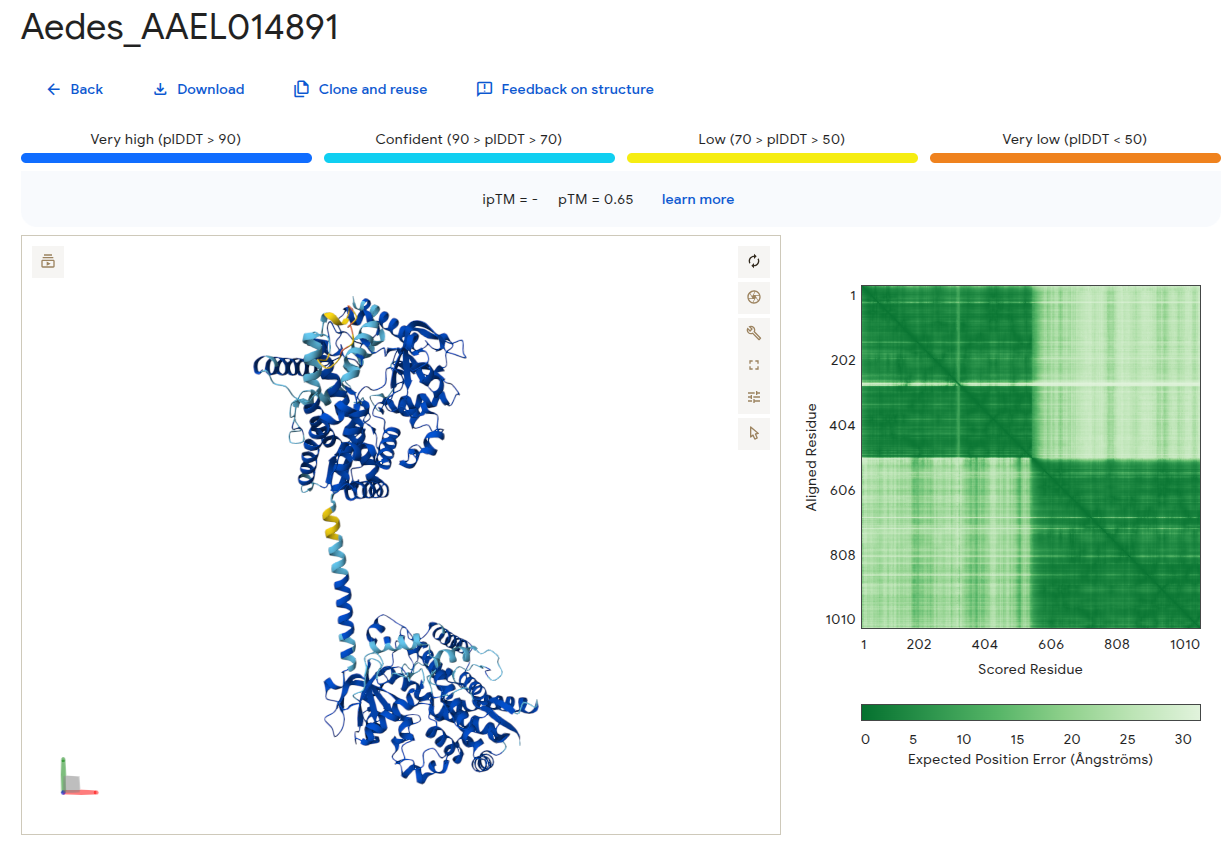


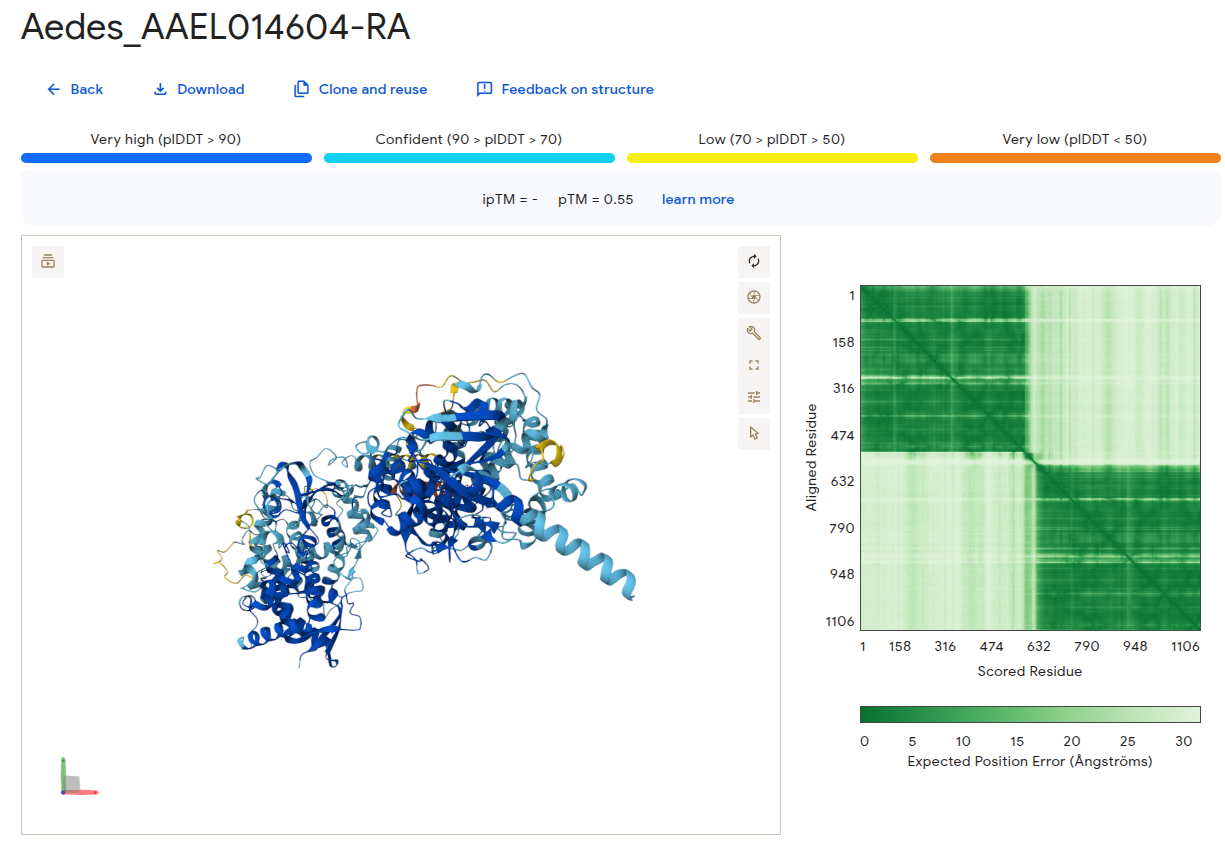


**Supplementary Figure S10. AlphaFold3 structure prediction and PAE plots for two mis-annotated Cytochrome P450 genes from *Aedes aegypti*.**

**Supplementary Table S1. Analysis of GO Terms linked to multi-copy genes.** Summary GO terms were found using rrvigo and example genes or types extracted from EBI-QuickGO portal (accessed May 2025).

| **Summary GO Term Name** | **GO Term Representative (Parent)** | **Example gene family or type** | **Literature supports single or Multi-copy?** | **Publication** |
| --- | --- | --- | --- | --- |
| Serine-type endopeptidase activity | GO:0004252 | Serine peptidase | Multi-copy | [2] |
| Oxidoreductase activity, acting on paired donors, with incorporation or reduction of molecular oxygen | GO:0016705 | Cytochrome P450 | Multi-copy | [3] |
| Iron ion binding | GO:0005506 | Cytochrome P450 | Multi-copy | [3] |
| Heme binding | GO:0020037 | Cytochrome P450 | Multi-copy | [3] |
| Glutathione transferase activity | GO:0004364 | Glutathione S-transferase | Multi-copy | [4] |
| UDP-glycosyltransferase activity | GO:0008194 | UDP-glucosyltransferase | Multi-copy | [5] |
| Protein heterodimerization activity | GO:0046982 | Histone-fold | Unclear, too broad to assess |  |
| DNA Binding | GO:0003677 |  | Unclear, too broad to assess |  |
| Transmembrane transporter activity | GO:0022857 | ABC Transporter | Multi-copy | [6] |
| Olfactory receptor activity | GO:0004984 | Olfactory receptor gene | Multi-copy | [7] |

**Supplementary Table S2. Counts of potential mis-annotation cases of Helixer in comparison to an independent annotation of the *Daucus carota* genome.** The genome annotations from RefSeq, CarrotOmics and Helixer for the Daucus carota genome were filtered to keep the longest isoform and the protein sequences used to conduct a Reciprocal Best Hit with mmseqs. The ratios of the best hits were used to assign potential mis-annotation cases.

| **Ratio Category** | **RefSeq vs Helixer** | **RefSeq vs CarrotOmics** | **Interpretation** |
| --- | --- | --- | --- |
| 1:1 match | 28,235 | 26,271 | Genes same length |
| 1:2 fusion | 188 | 70 | Second annotation fuses two RefSeq genes |
| 1:3 fusion | 13 | 5 | Second annotation fuses three RefSeq genes |
| 1:4 fusion | 1 | - | Second annotation fuses four RefSeq genes |
| 2:1 split | 158 | 284 | RefSeq fuses two genes from second annotation |
| 3:1 split | 16 | 37 | RefSeq fuses three genes from second annotation |
| 4:1 split | 2 | 7 | RefSeq fuses four genes from second annotation |
| other | 1,178 | 1,284 | Other length ratios |

**Supplementary Table S3: Counts and proportion of chimeric mis-annotations identified as a proportion of the total number of genes.**

| **Organism** | **Total Confirmed Chimeras** | **Candidate number of new genes** | **Total Genes** | **Proportion of total** |
| --- | --- | --- | --- | --- |
| Achroia_grisella | 27 | 56 | 14282 | 0.19% |
| Anolis_sagrei | 8 | 16 | 22331 | 0.04% |
| Anser_cygnoides | 9 | 18 | 25167 | 0.04% |
| Apis_mellifera | 24 | 51 | 12295 | 0.20% |
| Apteryx_mantelli | 12 | 24 | 20890 | 0.06% |
| Bactrocera_neohumeralis | 24 | 52 | 15890 | 0.15% |
| Bactrocera_tryoni | 18 | 37 | 15374 | 0.12% |
| Daucus_carota | 35 | 72 | 38174 | 0.09% |
| Drosophila_grimshawi | 23 | 61 | 13657 | 0.17% |
| Emydura_macquarii | 12 | 25 | 24567 | 0.05% |
| Euphorbia_lathyris | 11 | 23 | 33825 | 0.03% |
| Galleria_mellonella | 21 | 42 | 14749 | 0.14% |
| Gastrolobium_bilobum | 20 | 40 | 29617 | 0.07% |
| Heteronotia_binoei | 15 | 30 | 24267 | 0.06% |
| Lolium_rigidum | 35 | 71 | 56848 | 0.06% |
| Lycium_ferocissimum | 16 | 32 | 34894 | 0.05% |
| Miscanthus_floridulus | 24 | 48 | 80664 | 0.03% |
| Musa_acuminata | 18 | 36 | 120108 | 0.01% |
| Musca_vetustissima | 22 | 48 | 12930 | 0.17% |
| Oryza_sativa_japnoica | 17 | 35 | 37469 | 0.05% |
| Pezoporus_wallicus | 5 | 10 | 17755 | 0.03% |
| Phragmites_australis | 27 | 54 | 43427 | 0.06% |
| Pseudorca_crassidens | 3 | 6 | 27599 | 0.01% |
| Quercus_suber | 18 | 37 | 38452 | 0.05% |
| Rattus_rattus | 6 | 12 | 25450 | 0.02% |
| Saccostrea_cucullata | 28 | 58 | 38071 | 0.07% |
| Saccostrea_echinata | 31 | 63 | 32845 | 0.09% |
| Spodoptera_frugiperda | 32 | 71 | 16712 | 0.19% |
| Ylistrum_balloti | 9 | 19 | 20847 | 0.04% |
| Zophobas_morio | 55 | 112 | 26556 | 0.21% |

**Supplementary Table S4. Comparison of mis-annotated Glutathione S-Transferases (GSTs) between the RefSeq, Corn and Rice genomes of *Spodoptera frugiperda*.** Mis-annotated GSTs were initially identified for the RefSeq genome and annotation and the reciprocal best hit found for each in the *S. frugiperda* corn and *S. frugiperda* rice annotations using mmseqs easy-rbh. Chimeric designation was based off of visual inspection of the predicted-vs-aligned error plots produced from AlphaFold3 structure predictions of the protein sequences for each GST gene.

| **RefSeq annotation** | | | **Corn strain** | | | **Rice strain** | | |
| --- | --- | --- | --- | --- | --- | --- | --- | --- |
| *Spodoptera frugiperda* RefSeq annotation | Length (amino acids) | Chimeric designation | Matching gene | Length (amino acids) | AlphaFold3 Chimeric Clustering | Matching gene | Length (amino acids) | AlphaFold3 Chimeric Clustering |
| LOC118271633 | 409 | Double (2x) | SFRUCORN70000016687-PA | 358 | Double (2x) | SFRURICE0000023002-PA | 489 | Double (2x) |
| LOC118261724 | 465 | Double (2x) | SFRUCORN70000013771-PA | 1071 | At least 4x, potentially 5x | SFRURICE0000030251-PA | 1078 | At least 4x, potentially 5x |
| LOC118266848 | 450 | Double (2x) | SFRUCORN70000010054-PA | 451 | Double (2x) | SFRURICE0000005971-PA | 690 | Triple (3x) |
| LOC118270149 | 417 | Double (2x) | SFRUCORN70000020057-PA | 185 | Single (1x, non-chimeric) | SFRURICE0000001847-PA | 305 | Low confidence structure |

**Supplementary Table S5. Genomes and annotations for the genomes analysed in Mantica et al [1].** Comments provided if it was unable to access the same genome and annotation used by the authors in their analysis.

| **Species** | **Common Name [Code]** | **Assembly** | **Version** | **Source** | **Comment** |
| --- | --- | --- | --- | --- | --- |
| *Homo sapiens* | Human [Hsa] | hg38 | v88 | Ensembl | No mis-annotations identified by authors. **Excluded.** |
| *Mus musculus* | Mouse [Mmu] | mm10 | v88 | Ensembl | No mis-annotations identified by authors. **Excluded.** |
| *Bos taurus* | Cow [Bta] | bosTau9 | v99 | Ensembl | BosTau9 assembly matches “ARS-UCD1.2” assembly. |
| *Monodelphis domestica* | Opossum [Mdo] | monDom5 | v86 | Ensembl | Ensembl genome v86 doesn’t match provided assembly name (BROAD05 is up to v90, v91 onwards is monDom5). **Excluded.** |
| *Gallus gallus* | Chicken [Gga] | galGal6 | v99 | Ensembl |  |
| *Xenopus tropicalis* | Tropical clawed frog [Xtr] | XenTro9 | v101 | Ensembl |  |
| *Danio rerio* | Zebrafish [Dre] | danRer10 | v80 | Ensembl | Only .gtf available, conversion to gff3 carried out. |
| *Callorhinchus milii* | Elephant shark [Cmi] | 6.1.3 | v99 | Ensembl |  |
| *Strongylocentrotus purpuratus* | Sea urchin [Spu] | Spur_5.0 | v51 | Ensembl Metazoa |  |
| *Drosophila melanogaster* | Fruit fly [Dme] | dm6 | v26 | Ensembl Metazoa | No mis-annotations identified by authors. Version number invalid? **Excluded.** |
| *Aedes aegypti* | Yellow fever mosquito [Aae] | AaegL5 | v46 | Ensembl Metazoa |  |
| *Bombyx mori* | Domestic silk moth [Bmo] | ASM15162v1 | v45 | Ensembl Metazoa |  |
| *Tribolium castaneum* | Red flour beetle [Tca] | Tcas5.2 | v45 | Ensembl Metazoa |  |
| *Apis mellifera* | Honey bee [Ame] | Amel_4.5 | v35 | Ensembl Metazoa | Genome and annotation no longer available**. Excluded** |
| *Strigamia maritima* | Centipede [Sma] | Smar1 | v26 | Ensembl Metazoa |  |
| *Octopus bimaculoides* | California two-spot octopus [Obi] | ASM119413v2 | n/a | Ensembl Metazoa | Missing version number. **Excluded.** |
| *Branchiostoma lanceolatum* | Amphioxus [Bla] | 1 | n/a | Publication |  |
| *Episyrphus balteatus* | Marmalade hoverfly [Eba] | 2 | n/a | Publication |  |
| *Cloeon dipterum* | Mayfly [Cdi] | 3 | n/a | Publication | Matching genome and annotation data unavailable from source publication. **Excluded.** |
| *Blattella germanica* | Cockroach [Bla] | blager_OGSv1.2.1 | n/a | i5k (USDA) | Unable to match up GeneIDs from Supplementary Material to gtf from publication. **Excluded.** |

# Supplementary References

1. Mantica F, Iñiguez LP, Marquez Y, Permanyer J, Torres-Mendez A, Cruz J, et al. Evolution of tissue-specific expression of ancestral genes across vertebrates and insects. Nat Ecol Evol. 2024;8:1140–53.

2. Lawniczak MKN, Begun DJ. Molecular population genetics of female-expressed mating-induced serine proteases in Drosophila melanogaster. Molecular Biology and Evolution. 2007;24:1944–51.

3. Darragh K, Nelson DR, Ramírez SR. The Birth-and-Death Evolution of Cytochrome P450 Genes in Bees. Genome Biol Evol. 2021;13:evab261.

4. Yao P-H, Mobarak SH, Yang M-F, Hu C-X. Differential detoxification enzyme profiles in C-corn strain and R-rice strain of Spodoptera frugiperda by comparative genomic analysis: insights into host adaptation. BMC Genomics. 2025;26:14.

5. Ren C, Cao Y, Xing M, Guo Y, Li J, Xue L, et al. Genome-wide analysis of UDP-glycosyltransferase gene family and identification of members involved in flavonoid glucosylation in Chinese bayberry (Morella rubra). Front Plant Sci. 2022;13:998985.

6. Zhang X, Zhao Y, Zheng W, Nan B, Fu J, Qiao Y, et al. Genome-wide identification of ATP-binding cassette transporter B subfamily, focusing on its structure, evolution and rearrangement in ciliates. Open Biology. 2023;13:230111.

7. An X-K, Sun L, Liu H-W, Liu D-F, Ding Y-X, Li L-M, et al. Identification and expression analysis of an olfactory receptor gene family in green plant bug Apolygus lucorum (Meyer-Dür). Sci Rep. 2016;6:37870.
